# Determination of Flavonoids in Selected *Scleranthus* Species and Their Anti-Collagenase and Antioxidant Potential

**DOI:** 10.3390/molecules27062015

**Published:** 2022-03-21

**Authors:** Katarzyna Jakimiuk, Jakub W. Strawa, Sebastian Granica, Marcello Locatelli, Angela Tartaglia, Michał Tomczyk

**Affiliations:** 1 Department of Pharmacognosy, Faculty of Pharmacy with the Division of Laboratory Medicine, Medical University of Białystok, ul. Mickiewicza 2a, 15-230 Białystok, Poland; katarzyna.jakimiuk@umb.edu.pl (K.J.); jakub.strawa@umb.edu.pl (J.W.S.); 2 Microbiota Lab, Center for Preclinical Studies, Department of Pharmacognosy and Molecular Basis of Phytotherapy, Faculty of Pharmacy, Medical University of Warsaw, ul. Banacha 1, 02-097 Warsaw, Poland; sgranica@wum.edu.pl; 3 Department of Pharmacy, University of Chieti−Pescara “G. d’Annunzio”, 66100 Chieti, Italy; m.locatelli@unich.it (M.L.); angela.tartaglia@unich.it (A.T.)

**Keywords:** *Scleranthus perennis*, *Scleranthus annuus*, *c*-flavones, enzyme inhibition, collagenase, antioxidant

## Abstract

A new 5,7-dihydroxy-3′-methoxy-4′-acetoxyflavone-8-*C*-*β*-d-arabinopyranoside-2″-*O*-(4‴-acetoxy)-glucoside (**6**) and three known flavone *C*-glycosides—5,7,3′,4′-tetrahydroxyflavone-6-*C*-xyloside-8-*C*-*β*-d-glucoside (lucenin-1) (**7**), 5,7,3′-trihydroxyflavone-6-*C*-glucoside-8-*C*-*β*-d-glucoside (vicenin-2) (**8**), and 5,7,4′-trihydroxy-3′-methoxyflavone-6-*C*-*β*-d-glucopyranoside-8-*C*-*α*-arabinopyranoside (chrysoeriol-6-*C*-*β*-d-glucopyranoside-8-*C*-*α*-arabinopyranoside) (**9**)—were isolated from aerial parts of *Scleranthus perennis* L. (Caryophyllaceae). Their structures were determined through the use of comprehensive spectroscopic and spectrometric methods, and a method for the quantification of the major constituents of *S. perennis* and *S. annuus* L. was developed. Furthermore, the anti-collagenase and antioxidant activities of all isolated compounds obtained from extracts and fractions from both *Scleranthus* species were evaluated. The highest percentage of collagenase inhibition (at 400 µg/mL) was distinguished for methanolic extracts (22.06%, 32.04%) and ethyl acetate fractions (16.59%, 14.40%) from *S. annuus* and *S. perennis*. Compounds **6**–**9** displayed moderate inhibitory activity, with IC_50_ values ranging from 39.59–73.86 µM.

## 1. Introduction

*Scleranthus perennis* L. and *Scleranthus annuus* L., belonging to the Caryophyllaceae family, are endemic species to Europe, North Africa, and Western Asia [1,2]. Only a few studies have reported on the phytochemical description of these plants. To date, the allocated substances previously isolated from *S. annuus* were mostly phenolic acids and phytoecdysteroids, as well as flavonoids [3,4]. Our previous phytochemical study has shown the presence of new flavonoid *C*-glycosides in aerial parts of *S. perennis* (scleranthoside A–C), as well as a paenonside derivative [5]. As part of an ongoing investigation on *S. perennis* (or perennial knawel), we carried out a chemical study on the ethyl acetate and butanol fractions from perennial knawel, resulting in the isolation and structural characterization of the next four flavonoid *C*-glycosides on the basis of extensive spectroscopic and spectrometric analysis, including 1D and 2D NMR (COSY, HSQC, and HMBC) and ESI-MS spectra. Furthermore, the chemical descriptions of the polyphenolic compounds, as well as qualitative and quantitative validated analysis of extracts and fractions from both *S. perennis* and *S. annuus*, make them important starting materials for assessment of the chemotaxonomic characteristics of the *Scleranthus* genus.

Flavonoids possess topical anti-inflammatory and anti-aging potentials, as was shown in both in vitro and in vivo investigations [6]. It is well known that plants containing a variety of natural antioxidants can be assumed as candidates for moderating the effects of the skin-aging process by limiting the biochemical consequences of oxidation [7]. The main role of ROS (reactive oxygen species) is to exterminate and damage attacking micro-organisms and degrade destroyed tissue structures. Nonetheless, imprecise targeting of ROS may provoke oxidative stress in surrounding healthy cells, leading to an intensification of pathological processes. The excessive release of ROS occurs in the pathogenesis of numerous human skin diseases, including age-related disorders [8]. The ECM (extracellular matrix) is the part of the skin composed of, inter alia, proteins including collagen or elastin, and its degradation is tied with the skin-aging process [9]. Many studies have deliberated over the elevated level of some matrix metalloproteinases (MMP), such as collagenase or elastase, in inflamed and photoaged skin [10,11,12]. MMP decompose dermal matrix proteins, such as collagen, thus contributing to skin injuries and wrinkle formation. Consequently, factors with the ability to inhibit collagenase or elastase activity have an advantageous impact for supporting skin health, through the prevention of dermal matrix degradation [13]. In this respect, continuing the search for anti-collagenase substances and the most active components modulating the enzyme activity, a comparison between crude extracts and fractions obtained by chromatographic separation from both *S. perennis* and *S. annuus*, as well as pure standards, was carried out.

## 2. Results and Discussion

### 2.1. Isolation and Identification of Flavone C-Glycosides (***6**–**9***)

**Figure 1:**
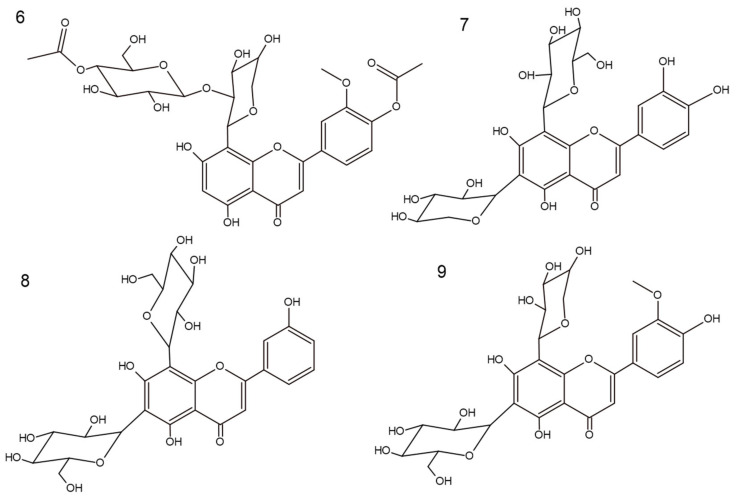
Chemical structures of compounds **6**–**9**.

In our previous research, one phenolic derivative (**5**) and four flavone derivatives (**1**–**4**) were isolated and fully identified [5]. As a result of ongoing exhaustive multi-step chromatographic isolation processes, one (**6**) and three (**7**–**9**) chromatographically homogeneous unknown compounds were isolated from the ethyl acetate (**SP5**) and butanol (**SP6**) fractions, respectively (Figure 1). The identification of those compounds was executed based on products of acid hydrolysis [5] and spectroscopic methods, such as ultraviolet (UV) spectroscopy, nuclear magnetic resonance (NMR), and mass spectrometry (MS).

#### 2.1.1. 5,7-Dihydroxy-3′-methoxy-4′-acetoxyflavone-8-*C*-*β*-d-arabinopyranoside-2″-*O*-(4‴-acetoxy)-glucoside (**6**)

**Figure 2:**
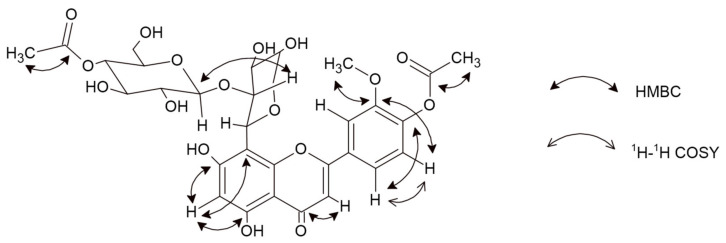
Important ^1^H–^1^H COSY and HMBC correlations for compound **6**.

Compound **6** was received as a yellow amorphous powder. Based on the HRESIMS ion peak at *m*/*z* 679 [M + H]^+^, the molecular formula of C_31_H_34_O_17_ was determined. Its UV spectrum showed absorption maxima at 263 and 287 nm, indicating the characteristic pattern of flavones. Sodium acetate (NaOAc) is used with flavones containing a 4′-hydroxyl group, where the band I appears as a peak similar to that observed with sodium methoxide (NaOMe) (304 nm and 301 nm, respectively). Furthermore, in the presence of NaOMe, a bathochromic shift of 17 nm indicated that C4′ is substituted. A bathochromic shift of 12 nm in the presence of NaOAc/H_3_BO_3_ indicated a B-ring containing an ortho-substitution pattern. Additionally, shifts of 14 nm (in the presence of NaOAc) and 31 nm (in the presence of AlCl_3_/HCl) signify free hydroxyl groups at C5 and C7 [14]. The ^1^H and ^13^C NMR data showed similarities to scleranthoside C [5], except for a set of signals assignable to an arabinose group. The appearance of one proton singlet at *δ* 6.23 in the ^1^H spectrum displays a tri-substituted pattern of A-ring. The absence of signals in the ^13^C spectrum points out a moiety in a C8. In the HMBC experiment, the proton at *δ* 6.23 exhibited a correlation with C5 (*δ* 161.39) and C8 (*δ* 104.59), which led to the assignment of this proton to position H-C6 [15,16]. Carbons in an A-, C-, and B-rings were assigned based on meticulous analysis of the ^13^C spectrum, supported by 2D NMR correlations and previous identification of *C*-flavone glycosides [5]. Thus, based on COSY correlation, the signals at *δ* 6.95 (1H, d, *J* = 7.78 Hz) and *δ* 7.50 (1H, d, *J* = 7.78 Hz) were assigned to H-C5′ and H-C6′, respectively. Further, the carbon signals at *δ* 150.46 and *δ* 148.06 correspond to an ortho-coupling 3′,4′-deoxidized B-ring [17]. The inherence of the double bond appears at *δ* 182.74 in the ^13^C NMR spectrum, which corresponds to the C4. The HMBC data showed that the C4 signal was combined with the proton signal at *δ* 6.58 allocated to H-C3. The presence of a methyl group was concluded from ^1^H (*δ* 4.03) and ^13^C NMR (*δ* 55.32) and located at C3′ based on HMBC correlation. Furthermore, two acetoxy groups in the structure were revealed by a chemical shift of the –CH_3_ group in the ^1^H NMR spectrum as singlets at *δ* 1.97 and *δ* 2.02, as well as in the ^13^C NMR spectrum for acetoxycarbonyl carbon at *δ* 170.72 and *δ* 171.47 and acetoxymethyl carbon at *δ* 19.31 and *δ* 19.58 [5,17]. This conclusion was further maintained by the HSQC and HMBC correlations (see Figure 2 and Figures S5–S7 in the Supplementary Materials).

Two anomeric proton signals at *δ* 5.16 (d, 1H, *J* = 9.29 Hz) and *δ* 4.31 (d, 1H, *J* = 7.53 Hz) indicated the presence of two sugar groups. The interglycosidic linkages of **6** were determined by analysis of the HMBC data. Based on the chemical shifts of the individual saccharides, one of them was *β*-d-glucose (*δ* 2.93–3.19) and the other was *β*-d-arabinopyranoside (*δ* 3.90–4.09) [5,17]. The arabinose residue was assigned to position 8 based on cross-peaks in HMBC correlations (*δ* 104.59 and *δ* 5.19). Moreover, a signal occurring in the ^1^H NMR spectrum at *δ* 4.31 (d, 1H) with J-coupling value of 7.53 Hz indicated an *O*-glycoside link. Through comparison of these data with the reported Rf value (0.55) of products of acid hydrolysis, the terminal sugar was classified as glucose. These data, together with the retro-Diels–Alder re-shuffling in a positive ESI-MS fragmentation pattern—637 [M + H-Ac], 475 [M + H-hex-Ac], 343 [M + H-hex-Ac-pent], and 313 [M + H-hex-Ac-pent-OCH_3_] (Figure S1)—suggested the presence of acetylation of hexose moiety, as well as methylation and acetylation of the B-ring. Thus, the structure of **6**, which is a new natural product, was established as 5,7-dihydroxy-3′-methoxy-4′-acetoxyflavone-8-*C*-*β*-d-arabinopyranoside-2″-*O*-(4‴-acetoxy)-glucoside and named scleranthoside D (Figure 1).

#### 2.1.2. 5,7,3′,4′-Tetrahydroxyflavone-6-*C*-xyloside-8-*C*-*β*-d-glucoside (Lucenin-1) (**7**)

Compound **7** was obtained as a yellow amorphous powder. Based on the HRESIMS ion peak at *m*/*z* 581 [M + H]^+^, the molecular formula of C_26_H_28_O_17_ was determined. Based on UV spectra, **8** was assigned to the flavone derivatives and showed a bathochromic shift of band I in the presence of NaOAc/H_3_BO_3_ (29 nm), indicating an ortho-dihydroxyl group in the B-ring (luteolin derivative) [14]. This was confirmed by ^13^C NMR data, which showed signals for 26 carbons. Comparing ^1^H NMR data of **6** and **7** showed two signals from glycosyl moieties at *δ* 5.09 (d, 1H, *J* = 9.79 Hz) and *δ* 4.98 (d, 1H, *J* = 9.79 Hz), indicating substitutions at C8 and C6, respectively. Furthermore, the mass fragmentation pattern in positive mode (180 V) was as follows: 563, 545, 527, and 497. This indicated a typical system for 6,8-*C*-glycosides [18,19]. The ^13^C NMR and ^1^H NMR signals of the compound were consistent with literature data [20,21]. Hence, the structure of **7** was established as 5,7,3′,4′-tetrahydroxyflavone-6-*C*-xyloside-8-*C*-*β*-d-glucoside (lucenin-1) (Figure 1).

#### 2.1.3. 5,7,3′-Trihydroxyflavone-6-*C*-glucoside-8-*C*-*β*-d-glucoside (Vicenin-2) (**8**)

Compound **8**, a yellow amorphous powder, exhibited a predominant ion peak at *m*/*z* 595 [M + H]^+^ in positive mode by HRESIMS, corresponding to the molecular formula C_27_H_30_O_15_. According to UV spectra, **8** is different from **7** by a lack of hydroxylation at C4′. This conclusion was supported by observations of a similar value of band I appearing with NaOAc/H_3_BO_3_ (347 nm) and in MeOH (345 nm) [14]. As described above, in ^1^H NMR spectra, two signals for saccharides bonds were observed—*δ* 5.08 (d, 1H, *J* = 9.79 Hz) and *δ* 4.98 (d, 1H, *J* = 9.79 Hz)—pointing out C6 and C8 as their binding sites. Furthermore, in the positive ESI mass spectrum, the fragments *m*/*z* 575, 503, 473, 383, and 353 provided the location and bond type for two hexose moieties [22]. Combining the information from the previous literature, fragmentation information, and spectral data, compound **8** was established as 5,7,3′-trihydroxyflavone-6-*C*-glucoside-8-*C*-*β*-d-glucoside (vicenin-2) (Figure 1) [21,22].

#### 2.1.4. 5,7,4′-Trihydroxy-3′-methoxyflavone-6-*C*-*β*-d-glucopyranoside-8-*C*-*α*-arabinopyranoside (Chrysoeriol-6-*C*-*β*-d-glucopyranoside-8-*C*-*α*-arabinoside) (**9**)

Compound **9**, isolated as a yellow amorphous powder, showed an [M + H]^+^ ion at *m*/*z* 595 in its HRESIMS spectrum, corresponding to the molecular formula C_27_H_30_O_15_. The UV spectra are typical of flavone derivatives with ortho-hydroxylation (bathochromic shift of band I in the presence of NaOAc/H_3_BO_3_) [14]. Moreover, the occurrence of signals *δ* 56.77 in ^13^C spectra and *δ* 3.97 in ^1^H spectra are characteristic of –CH_3_. HMBC correlation of *δ* 3.97 with *δ* 149.37 indicated that C3′ was methylated [23]. In the positive ESI-MS spectra of **9**, the pattern observed was in agreement with those of *C*-glycoside derivatives: 577, 475, 409, 385, and 355 [22]. The ^1^H and ^13^C NMR data of compound **9** were consistent with literature data [24]. Thus, the structure was established as 5,7,4′-trihydroxy-3′-methoxyflavone-6-*C*-*β*-d-glucopyranoside-8-*C*-*α*-arabinopyranoside (chrysoeriol-6-*C*-*β*-d-glucopyranoside-8-*C*-*α*-arabinopyranoside) (Figure 1), which was previously isolated from the genus Silene (Caryophyllaceae family) [24].

### 2.2. Phytochemical Analysis of Extracts ***SP1**–**SP3***, ***SA1**–**SA3*** and Fractions ***SP4**–**SP6***, ***SA4**–**SA6***

HPLC-PDA-MS^n^ analysis (Table 1) of all 12 extracts and fractions revealed the presence of 24 different phenolics, based on their characteristic UV and MS^n^ spectra (Figures S8–S19), which were assigned to the groups of phenolic derivatives (I, II) or flavonoid derivatives (III–XXIV). The glycosidic derivatives present in the analyzed samples were characterized by the specific loss of a substituted or unsubstituted sugar neutral molecule, which indicates the presence of a *C*-glycosidic bond, as well as an *O*-glycosidic linkage. The second characteristic group of compounds are flavone glycosides, substituted in two places with C-bonds. The occurrence of the specific [M-120-H]^−^/[M-90-H]^−^ ion pairs indicated that they were flavone 6,8-di-*C*-hexoses.

Besides compounds identified in the HPLC analysis, many other phytochemicals could be responsible for the biological effects of the investigated species. In this study, the values of total phenolic, phenolic acid, and tannin contents were determined, which are provided in Table 2. Total phenolics content of *S. perennis* extracts and fractions varied from 99.20 to 240.00 mg Peq/g, while those in extracts and fractions of *S. annuus* ranged from 95.72 to 217.20 mg Peq/g. According to the obtained results, **SA5** (11.34 mg CAeq/g) and **SA6** (9.19 mg CAeq/g) contained the highest total phenolic acids content, while the total tannins content was elevated in **SP2** (20.16 mg Peq/g).

It has been disputed that total bioactive compounds content assays provide limited insight into the phytochemical composition, as compared with complete extract or fraction profiles. At this point, our ongoing study is concentrated on the quantification of compounds isolated from the aerial parts of the two *Scleranthus species* (**SA1**–**SA6**, **SP1**–**SP6**) using HPLC. As reported in Table 3, compound **3** (59.10 ± 0.08 mg/g of extract) was quantified as a dominant substance in **SP5**. The quantification results showed that **SA5** and **SP5** are rich sources of flavonoids. Considering the fact that compounds **7**, **8,** and **9** were not detected in **SP5**, they could be assumed as markers differentiating **SA5** from **SP5**. Furthermore, the phenolic derivative (**5**) was present only in two samples from *S. annuus* (**SA5**, **SA6**), while only one fraction from *S. perennis* (**SP4**) did not contain compound **5**.

### 2.3. Antioxidant Activities of ***SP1**–**SP6***, ***SA1**–**SA6***, and Compounds ***1**–**9***

DPPH and ABTS assays are two methods used to estimate radical scavenging activity (RSA). The RSA for **SP1**–**SP6**, **SA1**–**SA6**, and compounds **1**–**9** are provided in Table 4. The radical scavenging activity for DPPH ranged from 64.07–386.59 µM Teq (Trolox equivalent) for all extracts and 6.69–409.16 µM Teq for compounds. In the ABTS assay, it ranged from 67.94–459.29 µM Teq, and from 169.27–765.16 µM Teq for extracts and compounds, respectively. Therefore, fractions (**SP4**–**SP6**, **SA4**–**SA6**) possessed both higher DPPH and ABTS activities than extracts (**SP1**–**SP3**, **SA1**–**SA3**). Among all tested compounds, **1**, **7**, and **9** were found to be progressive antiradical agents. On the other hand, **SP2** and compounds **3**, **4**, and **6** exhibited the lowest RSA values in the group of all tested samples. The reducing power of ions is another valid mechanism involved in the antioxidant pathway. To this end, FRAP and CUPRAC assays were evaluated at this point (see Table 4). As was observed in the DPPH and ABTS assays, fractions possessed higher-level antioxidant activity than extracts, both in FRAP and CUPRAC methods. The fractions were graded from the strongest to weakest, as follows: SP5 > SP6 > SA5 > SA6 in the CUPRAC assay, and SA6 > SA5 > SP5 > SP6 in the FRAP assay. The greatest activity of all compounds isolated from *S. perennis* was observed in compounds **1** (39.25 mM Fe^2+^/mL; 581.32 µM Teq) and **7** (45.30 mM Fe^2+^/mL; 116.16 µM Teq), while the weakest were compounds **3** (2.37 mM Fe^2+^/mL; 67.30 µM Teq) and **6** (3.05 mM Fe^2+^/mL; 18.57 µM Teq).

Based on the results outlined herein, the studied fractions containing the highest total phenolic, flavonoid, and phenolic acid contents (Table 2 and Table 3) likewise showed the highest antioxidant activity, which is in the agreement with the available literature [8,25,26]. Moreover, the improvement of the antioxidant capacity of all tested compounds can be associated with the C4′ hydroxyl group, as shown in compounds **1** and **7,** and the degradation of their activity with methylation or acetylation (see compounds **3**, **4**, and **6**) [27].

### 2.4. In Vitro Anti-Collagenase Inhibition

In a previous study, compounds **1**–**5** were investigated for their anti-collagenase activity [5]. The collagenase inhibition potential of compounds **6**–**9** isolated and identified during the present work was also established for the first time. Table 5 shows the activities of compounds **6**–**9** and their respective IC_50_ values.

Moreover, the collagenase inhibition activity of crude extracts (**SP1**–**SP3**, **SA1**–**SA3**) and fractions (**SP4**–**SP6**, **SA4**–**SA6**) from both *S. perennis* and *S. annuus* was assessed. The effect of their anti-collagenase potential, expressed as the percentage of anti-collagenase activity at 400 µg/mL, is summarized in Table 6.

Among the twelve extracts, all of them exhibited restrained anti-collagenase activity. At 400 µ/mL, **SA1** showed 32.04 ± 0.45% inhibition, which was stronger than that of the other extracts. The inhibition activity (>15%) of extracts can be presented, in decreasing order, as follows: **SA1** > **SP1** > **SA4** > **SA5** > **SP5**. On the other hand, butanol extracts from both *S. annuus* (**SA6**) and *S. perennis* (**SP6**) exhibited minimum percentage inhibition of 7.11 ± 0.17% and 2.39 ± 0.07%, respectively. Meanwhile, **SP2** and **SP4** showed low activity (>7%). As has been described in previous studies, a high content of flavonoid compounds is not necessarily related to the collagenase inhibitory effect [28]. In the ongoing search for anti-collagenase substances, the compounds newly isolated from *S. perennis* (**6**–**9**) were tested. In agreement with our previous statement [5], flavonoid *C*-glycosides displayed moderate activity, with IC_50_ ranging from 39.59 ± 1.21 to 73.86 ± 1.03 µM. Previous studies have implied that topically applied flavonoids may protect against collagen degradation and contribute to the prevention of its degradation in inflamed as well as in photoaged skin. Nevertheless, this potential activity needs to be additionally explained, in order to clarify their effect on the skin through topical application [6].

## 3. Materials and Methods

### 3.1. General Experimental Procedures

All used solvents for extraction and fractionation process, reagents for antioxidant and anti-collagenase assays, and equipment are provided in the Supplementary Materials. Furthermore, luteolin (purity > 96%), as well as compounds **1**–**5**, were isolated in the Department of Pharmacognosy, Medical University of Białystok [5,29].

### 3.2. Plant Material

The aboveground parts of *S. perennis* and *S. annuus* were collected from plants occurring in their natural habitat within the area of Bialystok from August through September in 2018 and 2019. The plant material was dried in a shaded and ventilated area. Stored *S. perennis* and *S. annuus* samples were recognized based on the scientific botanical bibliography [30]. Herbarium specimens were given voucher numbers (no. SP-18041 and no. SA-18042) and preserved in the Herbarium of the Department of Pharmacognosy at the Medical University of Białystok, Białystok, Poland.

### 3.3. Preparation of Extracts ***SP1**–**SP3***, ***SA1**–**SA3*** and Fractions ***SP4**–**SP6***, ***SA4**–**SA6***

The raw plant materials from *S. annuus* and *S. perennis* (10 g per sample) were powdered and subsequently extracted using an ultrasonication bath (5 × 30 min). All extractions were performed using 100 mL of one of the following solvents: MeOH (**SP1**, **SA1**), 50% MeOH (**SP2**, **SA2**), or H_2_O (**SP3**, **SA3**). After the evaporation of solvents under reduced pressure (temp. 30 ± 2 °C), residues were suspended in water and lyophilized. The following amounts of the samples were obtained: **SP1**, 1276 mg; **SA1**, 1970 mg; **SP2**, 880 mg; **SA2**, 1921 mg; **SP3**, 655 mg; and **SA3**, 2181 mg. Purification of the plant material (100 g) was conducted through the continuous extraction method using petrol (1.5 L × 20 h), and then CHCl_3_ (chloroform; 1.5 L × 20 h) in a Soxhlet apparatus. The purified raw material was exhaustively extracted with MeOH (20 × 1.5 L) and 50% MeOH (3 × 1.5 L) and H_2_O (1 × 1.5 L) for 1 h each time. After they were obtained, all extracts were combined, evaporated to dryness, and precipitated with water. Extracts obtained from both *S. annuus* and *S. perennis* were exhaustively fractionated by liquid–liquid extraction with different solvents of increasing polarity: CHCl_3_ (30 × 200 mL), Et_2_O (**SP4**, **SA4**; 35 × 200 mL), EtOAc (**SP5**, **SA5**; 100 × 200 mL), and *n*-BuOH (**SP6**, **SA6**; 60 × 200 mL). All fractions were evaporated, dissolved in water, and finally lyophilized using a freeze dryer. The six fractions were obtained in the following amounts: **SP4**, 850 mg; **SA4**, 100 mg; **SP5**, 821 mg; **SA5**, 632 mg; **SP6**, 563 mg; and **SA6**, 452 mg.

### 3.4. Phytochemical Analysis of Extracts ***SP1**–**SP3***, ***SA1**–**SA3*** and Fractions ***SP4**–**SP6***, ***SA4**–**SA6***

#### 3.4.1. Determination of Total Phenol Content

Total phenol (TPC) content was examined using transformed Folin–Ciocalteu colorimetric analysis [26] by mixing 80 µL of extract or fraction solution (1 mg/mL), 80 µL of Folin–Ciocalteu (9:1 *v*/*v*) reagent, and 80 µL of 10% Na_2_CO_3_ (sodium carbonate). After 1 h of incubation at 25 °C, absorbance at 630 nm was measured. TPC is expressed as the equivalent of pyrogallol from the calibration curve. Each assay was repeated in triplicate.

#### 3.4.2. Determination of Total Tannin Content

Determination of total tannin content (TTC) was evaluated using the difference between values of TPC, as described in the European Pharmacopoeia, 10th Edition [31]. In short, 1 mL of sample (1 mg/mL) was shaken with 10 mg of leather powder and then percolated. Then, 80 µL of the filtrate was mixed with 80 µL of Folin–Ciocalteu (9:1 *v*/*v*) reagent and 80 µL of sodium carbonate (10%). After 1 h, the absorbance was measured at 630 nm.

#### 3.4.3. Determination of Phenolic Acid Content

Total phenolic acid content (TPAC) was determined using a previously described method with Arnov’s reagent [32]. Briefly, 30 µL of the sample (1 mg/mL) was mixed with 150 µL of distilled water, 30 µL of HCl (0.5 M), 30 µL of Arnov’s reagent, and 30 µL of NaOH (1 M). Absorbance was measured at 490 nm, and the values are expressed as equivalent of caffeic acid.

#### 3.4.4. Qualitative HPLC-MS^n^ Analysis

Screening of the metabolites in the extracts and fractions was performed using an Ultimate 3000 series HPLC system coupled with an Amazon SL (Bruker, Bremen, Germany) ion trap mass spectrometer. A C18 reversed-phase packing column with the security guard column (Kinetex XB-C18, 150 × 2.1 mm, 1.7 μm; Phenomenex, Torrance, CA, USA) was used for the separation. The column was thermostated at 45 ± 0.5 °C using a column oven. The acquisition of the UV–vis spectrum was set in the range of 190–600 nm, while the UV chromatogram was recorded at a wavelength of 270 nm for V and 348 nm for flavonoids. Gradient elution was performed using the mobile phase water–acetonitrile starting at 93:7 (*v:v*), both with 0.1% formic acid. After 3 min, there was an accumulation of solvent B to 10.5% in 6 min, 13% in 37 min, and 17.5% in 40 min. Then, the previous proportion was maintained for 90 min. The last two steps were followed by a linear gradient to 50% in 108 min and an isocratic elution in 2 min. Finally, it was equilibrated to the starting conditions for 10 min. The injection volume was 5.0 µL and the flow rate was 0.3 mL/min. The IT-MS^n^ conditions were as follows: gas flow of 12 L/min with a temperature of 325 °C, a nebulizer pressure of 45 psi, capillary voltage of 2500 V with nozzle voltage 1000 V for negative ion mode, and electrospray ionization (ESI) source in ionization mode.

#### 3.4.5. Quantitative Analysis

The stock solutions were made at a concentration of 1 mg in a final volume of 1 mL. Working solutions of mixed standards were in the concentration range of 0.05–10 μg/mL for **3** and 0.01–5 μg/mL for **5**, which were made through the dilution of stock solution in volumetric flasks with the mobile phase. Then, the standards were injected into the HPLC-PDA system in six concentration levels. The 12 samples (**SA1**–**SA6**, **SP1**–**SP6**), prepared by accurately weighing, were dissolved and diluted with mobile phase to final 2 mg/mL. Then, 5 µL was directly injected into the HPLC-PDA system. The selectivity of the elution method and the linearity of each calibration curve (R^2^) were taken into consideration for the accuracy of the method. The LOD (limits of detection) and LOQ (limits of quantification) of analytes were evaluated using the standard deviation of the response (SDa) and the slope (b) of calibration curves. Consequently, LOD and LOQ are expressed as 3.3 × SDa/b and 10 × SDa/b, respectively. The repeatability and reproducibility of the following methods were confirmed by intra-day and inter-day precision analyses. Considering the intra-day precision, three replicates for each analyte at a specific concentration were carried out; while, for the inter-day precision, nine replicates were conducted over three days [33]. These parameters are given only for **3** as a dominant flavonoid compound isolated from *S. perennis*, as well as for **5**, as a non-flavonoid compound with different absorption maxima (λ = 270 nm). All differences and linear regression parameters for the standard curve values are presented in Table 7.

### 3.5. Isolation of Flavone C-Glycosides (***6**–**9***)

To isolate the flavonoid compounds, air-dried samples of *S. perennis* (500 g) were pulverized and extracted exhaustively in a Soxhlet extractor, first with petrol and then with chloroform. Afterward, the samples were extracted with methanol (45 × 3 L), 50% methanol (10 × 3 L), and water (3 × 3 L) for 1 h each time. Combined extracts from *S. perennis* (108.38 g) were evaporated to dryness at 45 °C and fractionated by Sephadex LH-20 column chromatography using methanol, resulting in 33 fractions. The obtained fractions were combined, after TLC analysis, into 5 fractions (F_1_–F_5_). According to preliminary LC-MS analysis, fraction F_3_ was introduced for further investigation. It was dissolved in water and fractionated by liquid−liquid extraction with Et_2_O, EtOAc, and finally *n*-BuOH. After, to isolate the flavonoids, the ethyl acetate and n-butanol fractions were purified and lyophilized. The EtOAc (2 g) and BuOH (2 g) fractions were separated using preparative HPLC (0–35 min, 0–7% UPW-ACN, 20 mL/min) in order to receive compound 6 (5.32 mg) from EtOAc, and compounds 7 (7.65 mg), 8 (5.54 mg), and 9 (9.26 mg) from BuOH. The purified compounds (**6**–**9**) were subjected to spectral and chromatographical analyses in order to determine their full structural characteristics. Spectral measurements were performed using the UV–vis method with various complexing reagents and ^1^H, ^13^C, COSY, HSQC, and HMBC spectra in CD_3_OD. Sugars were identified based on a chromatographic product of acid hydrolysis. Optical rotation was conducted in DMSO. The final characteristics of all isolated compounds were also confirmed by product ion scan.

### 3.6. Identification of Compounds ***6**–**9***

5,7-dihydroxy-3′-methoxy-4′-acetoxyflavone-8-*C*-*β*-d-arabinopyranoside-2″-*O*-(4‴-acetoxy)-glucoside (6): yellow amorphous powder (mp.: 187.0–188.0 °C); [*α*]_D_ −29.0 (DMSO; c 0.1); HPLC rt, 100.03 min; HRESIMS *m*/*z* = 679.205 [M + H]^+^ (calculated for C_31_H_34_O_17_); UV λ_max_ nm: 263, 287; +NaOMe: 252, 304; +AlCl_3_: 249, 318; +NaOAc: 260, 301; +H_3_BO_3_: 263, 297); NMR spectral data (Bruker, Oxford, UK), ^1^H NMR (CD_4_OD, 400 MHz) H-C2′: *δ* 7.62 (s, 1H), H-C6′: *δ* 7.50 (d, 1H, *J* = 7.28), H-C5′: *δ* 6.95 (d, 1H, *J* = 7.28), H-C3: *δ* 6.58 (s, 1H), H-C6: *δ* 6.23 (s, 1H), H-C1″: *δ* 5.16 (d, 1H, *J* = 9.29), H-C1‴: *δ* 4.31 (d, 1H, *J* = 7.53), H-C4″: *δ* 4.18 (s, 2H), H-C4″: *δ* 4.09 (s, 2H), CH_3_: *δ* 4.03 (s, 3H), H-C2″: *δ* 3.90 (s, 2H), H-C3″: *δ* 3.78 (s, 2H), H-C6‴: *δ* 3.19 (s, 2H), H-C5‴: *δ* 3.17 (s, 2H), H-C3‴: *δ* 3.04 (s, 2H), H-C4‴: *δ* 2.95 (s, 2H), H-C2‴: *δ* 2.93 (s, 2H), OAc: *δ* 1.97 (s, 1H), OAc: *δ* 2.20 (s, 1H); 13C NMR (CD_4_OD, 400 MHz) C4: *δ* 182.74, Ac: *δ* 171.47, *δ* 170.72, C2: *δ* 164.83, C7: *δ* 163.11, C5: *δ* 161.39, C9: *δ* 155.59, C4′: *δ* 150.46, C3′: *δ* 148.06, C1′: *δ* 122.76, C6′: *δ* 120.45, C5′: *δ* 115.40, C2′: *δ* 109.73, C8: *δ* 104.59, C1‴: *δ* 104.08, C3: *δ* 102.87, C10: *δ* 102.53, C6: *δ* 99.43, C2″: *δ* 80.42, C3‴: *δ* 76.45, C1″: *δ* 74.72, C3″: *δ* 74.61, C5‴: *δ* 73.60, C2‴: *δ* 72.71, C5″: *δ* 71.38, C4‴: *δ* 69.20, C4″: *δ* 68.05, C6‴: *δ* 63.11, CH_3_: *δ* 55.32, Ac: *δ* 19.58, Ac: *δ* 19.32; see Figures S1–S7 in the Supplementary Materials.

5,7,3′,4′-tetrahydroxyflavone-6-*C*-xyloside-8-*C*-*β*-d-glucoside (lucenin-1) (7): yellow amorphous powder; HPLC rt, 17.44 min; HRESIMS *m*/*z* = 579.167 [M + H]^+^ (calculated for C_26_H_28_O_15_); UV λ_max_ nm: 263, 287; +NaOMe: 252, 304; +AlCl_3_: 249, 318; +NaOAc: 260, 301; +H_3_BO_3_: 263, 297); NMR spectral data, see [20].

5,7,3′-trihydroxyflavone-6-*C*-glucoside-8-*C*-*β*-d-glucoside (vicenin-2) (8): yellow amorphous powder; HPLC rt, 29.15 min; HRESIMS *m*/*z* = 595.184 [M + H]^+^ (calculated for C_27_H_30_O_15_); UV λ_max_ nm: 274, 345; +NaOMe: 285, 412; +AlCl_3_: 281, 362; +NaOAc: 283, 368; +H_3_BO_3_: 274, 247; NMR spectral data, see [34].

5,7,4′-trihydroxy-3′-methoxyflavone--6-*C*-*β*-d-glucopyranoside-8-*C*-*α*-arabinopyranoside (chrysoeriol-6-*C*-*β*-d-glucopyranoside-8-*C*-*α*-arabinopyranoside) (9): yellow amorphous powder; HPLC rt, 29.15 min; HRESIMS *m*/*z* = 595.184 [M + H]^+^ (calculated for C_27_H_30_O_15_); UV λ_max_ nm: 274, 349; +NaOMe: 285, 414; +AlCl_3_: 281, 360; +NaOAc: 283, 377; +H_3_BO_3_: 274, 282; NMR spectral data, see [24].

### 3.7. Antioxidant Activity

#### 3.7.1. DPPH Assay

Individual test compounds and extracts in methanol (130 µL) were added to 70 µL of a solution of DPPH (2,2-diphenyl-1-picrylhydrazyl). After 30 min of incubation, the absorbance was evaluated at 517 nm [26]. The determination was performed in triplicate and corrected to a blank sample (pure methanol). The radical scavenging activity of the tested samples was expressed as Trolox equivalents, calculated from the standard curve.

#### 3.7.2. ABTS Assay

Suppression of the production of the radical cation was measured using an antioxidant assay kit (CS0790). In brief, 10 µL of the test sample, 20 µL of myoglobin working solution, and 150 µL of ABTS working solution (containing ABTS substrate solution and 3% hydrogen peroxide) were mixed into a clear, flat-bottom 96-well plate. After 5 min of incubation, 100 µL of stop solution was added and absorbance was measured at 405 nm. The antioxidant concentration of tested samples was calculated using the equation obtained through linear regression of the Trolox standard curve.

#### 3.7.3. FRAP Assay

The chelating activity of the investigated substances toward ferrous ions was assessed using a ferric reducing antioxidant power assay kit. Briefly, 10 µL of each sample (**SP1**–**SP6**, **SA1**–**SA6**, **1**–**9**) was mixed with 190 µL of supplied reaction mix (containing FRAP Assay Buffer, FeCl_3_ solution, and FRAP Probe). After 1 h of incubation at 37 °C, the absorbance was measured at 594 nm. A blank solution containing 10 µL of MeOH instead of extract/compound solution was used. For further calculations, the ferrous standard curve was evaluated. Values are given as mM Ferrous equivalents. All tests were performed in triplicate.

#### 3.7.4. CUPRAC Assay

The reducing power of copper ions was evaluated using an antioxidant assay kit (MAK334). Briefly, 20 µL of sample and 100 µL of reaction mix were added into separate wells in a 96-well plate. After 10 min of incubation, the absorbance endpoint was measured at 570 nm. Total antioxidant capacity was determined using a standard curve evaluated for Trolox.

### 3.8. Anti-Collagenase Assay

The potential anti-collagenase activity was assessed as previously described [5]. In short, a solution including 25 µL of collagenase from *Clostridium histolyticum*, 25 µL of Tricine buffer, and 25 µL of various levels of the extracts (SP1–SP6, SA1–SA6) or compounds (**6**–**9**) were incubated at 37 °C. After 20 min, 75 µL of FALGPA was added. Then, absorbance was measured at 335 nm. The experiment was performed in 50 mM Tris buffer. The blank was evaluated using Tricine buffer, instead of sample, and the positive control was epigallocatechin gallate (EGCG). All measurements were conducted in triplicate and the IC_50_ for each sample was calculated. The percentage of collagenase inhibitory activity (CoInh) was calculated as follows:

Enzyme inhibition activity (%) = [1 − (B/S)] × 100%,

where B is the blank and S is the sample.

### 3.9. Statistical Analysis

All results obtained in the anti-collagenase test are represented as the mean ± standard deviation (SD) from at least three independent replicates. Statistical analysis was carried out using the GraphPad Prism 9 software (Trial, GraphPad Software, San Diego, CA, USA). Statistical differences and linear regression parameters for the standard curve were determined using ANOVA with corroboration of statistical significance. Calculations for regression parameters were conducted using the MS Excel 2019 software (Microsoft, Washington, DC, USA).

## 4. Conclusions

Plant metabolomics may deliver large data sets, which allow for a better understanding of the cellular processes occurring in plant organisms. The growing interest and development of metabolomics research in plants provide important indicators for the chemotaxonomic description of whole plant families. The identified compounds (**6**–**9**) isolated from *S. perennis* in this paper are newly found *C*-glycosylated flavones in *Scleranthus* plants. So far, flavone C-glycosides have only been reported in *S. uncinatus* Schur. [35]. It could be assumed that the two investigated species are chemically similar and partially clustered with other morphologically related species from the Caryophyllaceae family. Moreover, lucenin-1 (**7**), vicenin-2 (**8**), and chrysoeriol-6-*C*-*β*-d-glucopyranoside-8-*C*-*α*-arabinopyranoside (**9**) were previously isolated from Caryophyllaceae plants (e.g., *Stellaria* sp., *Spergularia* sp., *Silene* sp., or *Lychnis* sp.) [36]. Furthermore, to the best of our knowledge, compound 6 is a new chemical structure occurring in the whole plant kingdom, which extends the structural and chemical characterization of *Scleranthus* sp., as well as that of the Caryophyllaceae family. As phytochemical studies of both *S. perennis* and *S. annuus* have not been reported so far, we also performed qualitative and quantitative elucidation of all bioassay-guided extracts and fractions. All tested samples exhibited moderate anti-collagenase activity, and so, further biological models and the application of acylated *C*-diglycosyled flavones should be studied.

## Figures and Tables

**Table 1:** Qualitative analysis of **SP1**–**SP6** and **SA1**–**SA6** by liquid chromatography photodiode array detection mass spectrometry (LC-PDA-MS^n^).

Analyte	Rt (Min)	UV Spectra (Λ Max *Nm*)	-ESI–MS (*m*/*z*)	Fragmentation ^A^	Predicted Compounds
I	7.6	268, 302	459	293	Apiopaeonoside (**5**) ^I^
II	7.9	260, 300sh, 325	385	191	Quinic acid derivative
III	11.35	270, 345	579	459, 399, **369**, 313	Flavone *C*-hex-*C*-pent derivative
IV	12.8	257, 273, 348	579	**459**, 399, 369	Lucenin-1 (**7**) ^I^
V	17.51	270, 348	579	**459**	Flavone 6,8-di-*C*-glycoside derivative
VI	18.01	254, 270, 348	593	**473**	vicenin-2 (**8**) ^I^
VII	19.45	254, 270, 348	593	**473**, 383, 312	Flavone 6,8-di-*C*-glycoside derivative
VIII	21.86	255, 273, 348	593	**473**, 383, 312	Chrysoeriol-6-*C*-*β*-d-glucopyranoside-8-*C*-*α*-arabinoside (**9**) ^I^
IX	33.18	275, 348	593	**473**, 383	Flavone 6,8-di-*C*-glycoside derivative
X	35.23	270, 348	593	**473**, 383	Flavone 6,8-di-*C*-glycoside derivative
XI	40.13	265, 348	563	**473**, 383	Flavone *C*-hex-*C*-pent derivative
XII	41.64	270, 345	579	489, 399, **327**	Flavone *C*-hex-*C*-pent derivative
XIII	42.4	255, 270, 347	635	**413**, 308	Flavone *C*-hex-*C*-pent derivative
XIV	44.8	255, 270, 348	621	579, 531, 399, **327**	scleranthoside A (**1**) ^I^
XV	46.2	270, 345	677	**413**, 323	Flavone-*C*-pent-*O*-hex derivative
XVI	48.6	255, 270, 348	593	**413**, 323	scleranthoside B (**2**) ^I^
XVII	56.69	255, 270, 349	677	413	Flavone-*C*-pent-*O*-hex derivative
XVIII	58.47	254, 268, 348	677	545, 455, **413**, 322	scleranthoside C (**3**) ^I^
XIX	64.11	268, 348	635	413	Luteolin-8-*C*-pent-*O*-hex derivative
XX	66.86	268, 348	635	413	Luteolin-8-*C*-pent-*O*-hex derivative
XXI	74.55	253, 270, 348	677	**413**, 323	Luteolin-8-*C*-pent-*O*-hex derivative
XXII	75.98	252, 268, 348	677	413	scleranthoside D (**4**) ^I^
XXIII	90.87	270, 348	677	413	Luteolin-8-*C*-pent-*O*-hex derivative
XXIV	98.34	252, 268, 348	677	**635**, 545 455, 413	scleranthoside D (**6**) ^I^

^A^ bold, most abundant ion; hex, hexoside; pent, pentoside; ^I^ compounds **1**–**9** isolated from *S. perennis*.

**Table 2:** Phytochemical analysis of total phenolic (TPC), phenolic acid (TPAC), and tannin contents (TTC) of **SA1**–**SA6** and **SP1**–**SP6**.

Sample	TPC (Mg Peq/G Extract) ^A^	TPAC (Mg Caeq/G Extract) ^B^	TTC (Mg Peq/G Extract) ^A^
**SA1** **SP1**	103.16 ± 0.80 122.96 ± 1.96	7.20 ± 0.19 4.68 ± 0.18	8.64 ± 0.15 9.78 ± 0.98
**SA2** **SP2**	103.08 ± 1.84 107.76 ± 0.52	6.66 ± 0.18 6.03 ± 0.27	11.01 ± 0.98 20.16 ± 1.47
**SA3** **SP3**	95.72 ± 1.69 99.20 ± 0.40	6.12 ± 0.17 4.95 ± 0.12	7.35 ± 0.18 1.6 ± 0.15
**SA4** **SP4**	106.04 ± 2.71 165.16 ± 2.83	8.91 ± 0.27 5.58 ± 0.15	8.07 ± 1.35 12.57 ± 0.89
**SA5** **SP5**	217.20 ± 1.60 240.00 ± 2.01	11.34 ± 0.63 7.47 ± 2.80	6.3 ± 0.90 6.69 ± 0.97
**SA6** **SP6**	159.44 ± 1.40 161.32 ± 2.91	9.19 ± 0.54 5.67 ± 0.18	8.22 ± 0.45 1.74 ± 0.18

All data are represented as the mean with standard deviation from triplicate measurement; ^A^ expressed as pyrogallol equivalents (Peq); ^B^ expressed as caffeic acid equivalents (CAeq).

**Table 3:** Quantification of selected metabolites (**1**–**9**) in extracts and fractions (**SA1**–**SA6**, **SP1**–**SP6**).

Compound ^A^	SA1	SP1	SA2	SP2	SA3	SP3	SA4	SP4	SA5	SP5	SA6	SP6
**1**	BLQ	0.12 ± 0.01	0.20 ± 0.01	0.23 ± 0.01	ND	0.11 ± 0.01	BLQ	0.17 ± 0.01	1.61 ± 0.01	1.61 ± 0.01	0.57 ± 0.03	0.52 ± 0.04
**2**	0.56 ± 0.01	1.66 ± 0.01	1.10 ± 0.07	2.20 ± 0.11	0.73 ± 0.01	2.09 ± 0.07	0.12 ± 0.01	1.26 ± 0.01	5.08 ± 0.02	12.85 ± 0.01	1.28 ± 0.01	5.22 ± 0.01
**3**	0.36 ± 0.01	4.90 ± 0.05	0.94 ± 0.03	6.29 ± 0.13	0.62 ± 0.01	6.38 ± 0.06	0.34 ± 0.01	5.55 ± 0.01	2.03 ± 0.2	59.10 ± 0.08	0.32 ± 0.01	1.05 ± 0.02
**4**	BLQ	1.98 ± 0.02	BLQ	0.71 ± 0.01	ND	2.07 ± 0.01	0.24 ± 0.06	3.81 ± 0.01	8.14 ± 0.14	18.97 ± 0.08	ND	ND
**6**	1.05 ± 0.01	1.06 ± 0.02	0.58 ± 0.01	0.91 ± 0.04	0.68 ± 0.01	0.73 ± 0.01	0.36 ± 0.01	0.93 ± 0.01	3.79 ± 0.01	6.93 ± 0.04	BLQ	ND
**7**	0.72 ± 0.02	0.11 ± 0.01	0.56 ± 0.04	BLQ	0.46 ± 0.03	0.10 ± 0.01	ND	ND	0.25 ± 0.01	ND	3.40 ± 0.01	1.44 ± 0.02
**8**	0.19 ± 0.01	0.16 ± 0.01	0.16 ± 0.01	0.15 ± 0.01	0.13 ± 0.01	0.15 ± 0.01	ND	ND	0.28 ± 0.11	ND	0.90 ± 0.01	1.37 ± 0.01
**9**	0.11 ± 0.01	ND	BLQ	ND	BLQ	ND	ND	ND	0.33 ± 0.12	ND	0.45 ± 0.05	0.50 ± 0.01
Total	13.74 ± 0.1	17.4 ± 0.27	13.49 ± 0.18	18.69 ± 0.21	11.27 ± 0.05	18.97 ± 0.32	4.62 ± 0.16	17.6 ± 0.26	71.8 ± 3.1	136.13 ± 0.39	20.52 ± 0.78	21.03 ± 0.36
**5**	ND	1.37 ± 0.04	ND	2.11 ± 0.04	ND	1.08 ± 0.02	ND	ND	0.98 ± 0.12	0.83 ± 0.11	0.74 ± 0.04	7.74 ± 0.13

^A^ Content presented as mg/g of extract calculated in 3 equivalents for **1**–**4**, **6**–**9**; all data are represented as the mean with standard deviation from triplicate measurement; BLQ, below the limit of quantification; ND, not detected.

**Table 4:** Antioxidant activities of **SP1**–**SP6**, **SA1**–**SA6**, and compounds **1**–**9**.

Sample	DPPH ^A^ (µM Teq)	ABTS ^A^ (µM Teq)	FRAP ^B^ (mM Fe^2+^/mL)	CUPRAC ^A^ (µM Teq)
**SA1** **SP1**	193.16 ± 2.56 140.43 ± 2.76	215.74 ± 2.46 196.95 ± 1.23	2.02 ± 0.49 1.92 ± 0.43	7.14 ± 1.32 5.82 ± 0.32
**SA2** **SP2**	186.41 ± 1.93 64.07 ± 0.97	253.65 ± 3.72 67.94 ± 2.03	3.17 ± 0.66 1.57 ± 0.07	5.38 ± 0.76 9.34 ± 1.63
**SA3** **SP3**	118.49 ± 1.46 122.28 ± 1.83	425.69 ± 2.33 56.11 ± 2.13	3.30 ± 0.15 2.64 ± 0.33	13.29 ± 0.76 17.69 ± 1.96
**SA4** **SP4**	157.09 ± 0.97 273.53 ± 1.93	175.99 ± 2.03 443.96 ± 2.42	1.96 ± 0.53 4.65 ± 0.35	1.86 ± 0.32 5.33 ± 0.54
**SA5** **SP5**	354.32 ± 2.90 269.31 ± 0.97	577. 82 ± 3.51 311.45 ± 3.05	6.74 ± 0.98 6.73 ± 0.41	67.80 ± 1.32 97.26 ± 1.87
**SA6** **SP6**	386.59 ± 2.19 292.51 ± 2.03	459.29 ± 1.40 271.94 ± 1.68	7.50 ± 0.32 5. 78 ± 0.42	45.82 ± 1.01 70.44 ± 1.28
**1**	405.15 ± 3.12	765.16 ± 2.83	39.25 ± 1.42	581.32 ± 3.61
**2**	198.43 ± 2.40	188.57 ± 1.86	5.68 ± 0.68	114.56 ± 2.04
**3**	7.11 ± 0.59	198.57 ± 2.46	2.37 ± 0.36	67.30 ± 1.30
**4**	9.64 ± 0.82	302.05 ± 2.42	2.65 ± 0.47	44.22 ± 1.35
**5**	61.96 ± 1.90	444.23 ± 0.47	2.75 ± 0.11	48.46 ± 1.75
**6**	6.69 ± 0.97	169.27 ± 1.68	3.05 ± 0.25	18.57 ± 0.76
**7**	409.16 ± 2.03	796.61 ± 1.23	45.30 ± 2.49	116.16 ± 0.76
**8**	176.71 ± 0.97	369.24 ± 3.05	5.16 ± 0.96	27.15 ± 1.32
**9**	407.05 ± 1.83	586.69 ± 2.91	9.07 ± 0.45	18.20 ± 0.76

All data are represented as the mean with standard deviation from triplicate measurement; ^A^ expressed as Trolox equivalents (Teq); ^B^ expressed as Fe^2+^ equivalents.

**Table 5:** Anti-collagenase activity of compounds **6**–**9** and their respective IC_50_ values.

Compound	IC_50_ (µM)
**6**	39.59 ± 1.21
**7**	73.86 ± 1.03
**8**	53.37 ± 0.88
**9**	71.06 ± 0.97
EGCG ^A^	34.82 ± 0.53

All data are represented as the mean of IC_50_ values with standard deviation from triplicate measurements; ^A^ epigallocatechin gallate, positive control.

**Table 6:** Percentage of anti-collagenase activity of **SP1**–**SP6** and **SA1**–**SA6** (at 400 µg/mL).

Sample	Percentage of Inhibition (%) ^A^
**SA1** **SP1**	32.04 ± 0.45 22.06 ± 0.34
**SA2** **SP2**	10.90 ± 0.11 6.46 ± 0.09
**SA3** **SP3**	12.12 ± 0.41 11.13 ± 0.14
**SA4** **SP4**	19.42 ± 0.22 6.69 ± 0.10
**SA5** **SP5**	16.59 ± 0.19 15.40 ±0.32
**SA6** **SP6**	7.11 ± 0.17 2.39 ± 0.07

^A^ All data are represented as the mean of percentage values with standard deviation from triplicate measurements.

**Table 7:** Regression equation, linear range, limit of detection (LOD), limit of quantification (LOQ), accuracy, and precision obtained during optimization of the LC-PDA method.

Compound	Regression Equation ^A^	R^2^	Linear Range (µg/mL)	LOD (µg/mL)	LOQ (µg/mL)	Accuracy (%)	Precision (%) ^B^
**3**	y = 2374x + 63.119	0.9999	0.05–10	0.03	0.09	98.43 ± 3.70	1.3/1.95
**5**	y = 4563.2x − 28.098	0.9999	0.01–5	0.003	0.01	101.28 ± 3.95	0.97/1.23

^A^ The value for y corresponds to the peak area and x to the concentration, respectively; ^B^ expressed as RSD measured intra- and inter-day, respectively.

## Data Availability

Data are contained within the article and Supplementary Materials.

## Supplementary Materials

The following supporting information can be downloaded at: https://www.mdpi.com/article/10.3390/molecules27062015/s1. Figure S1: Product ion scan in positive mode of compound **6**; Figure S2: UV spectrum of compound **6**; Figure S3: ^1^H NMR spectrum (400 MHz) of compound **6** in CD_3_OD; Figure S4: ^13^C NMR spectrum (400 MHz) of compound **6** in CD_3_OD; Figure S5: ^1^H–^1^H COSY spectrum of compound **6** in CD_3_OD; Figure S6: HSQC spectrum of compound **6** in CD_3_OD; Figure S7: HMBC spectrum of compound **6** in CD_3_OD; Figure S8: UV–vis chromatogram (λ = 348nm) of separated compounds **1**–**4**, **6**–**9** obtained by LC-PDA-MS; Figure S9: UV–vis chromatogram (λ = 270 nm) of separated compounds **1**–**9** obtained by LC-PDA-MS. Figure S10: The qualitative assessment of **SA1**. UV–vis chromatogram (λ = 348 nm) obtained by LC-PDA-MS; Figure S11: The qualitative assessment of **SA2**. UV–vis chromatogram (λ = 348 nm) obtained by LC-PDA-MS; Figure S12: The qualitative assessment of **SA3**. UV–vis chromatogram (λ = 348 nm) obtained by LC-PDA-MS; Figure S13: The qualitative assessment of **SA4**. UV–vischromatogram (λ = 348 nm) obtained by LC-PDA-MS; Figure S14: The qualitative assessment of **SA5**. UV–vis chromatogram (λ = 348 nm and λ = 270 nm) obtained by LC-PDA-MS; Figure S15: The qualitative assessment of **SA6**. UV–vis chromatogram (λ = 348 nm and λ = 270 nm) obtained by LC-PDA-MS; Figure S16: The qualitative assessment of **SP1**. UV–vis chromatogram (λ = 348 nm and λ = 270 nm) obtained by LC-PDA-MS; Figure S17: The qualitative assessment of **SP2**. UV–vis chromatogram (λ = 348 nm and λ = 270 nm) obtained by LC-PDA-MS; Figure S18: The qualitative assessment of **SP3**. UV–vis chromatogram (λ = 348 nm and λ = 270 nm) obtained by LC-PDA-MS; Figure S19: The qualitative assessment of **SP4**. UV–vis chromatogram (λ = 348 nm) obtained by LC-PDA-MS.; Figure S20: The qualitative assessment of **SP5**. UV–vis chromatogram (λ = 348 nm and λ = 270 nm) obtained by LC-PDA-MS; Figure S21: The qualitative assessment of **SP6**. UV–vis chromatogram (λ = 348 nm and λ = 270 nm) obtained by LC-PDA-MS.

## Sample Availability

Samples of compounds **6**–**9** are available from the authors.

## Abbreviations

NMR	Nuclear magnetic resonance
COSY	Correlation spectroscopy
HSQC	Heteronuclear single quantum coherence
HMBC	Heteronuclear multiple bond correlation
UV	Ultraviolet radiation
UV–vis	Ultraviolet–visible spectroscopy
ESI	Electrospray ionization
IC_50_	Median inhibitory concentration
MS	Mass spectrometer
LC-MS	Liquid chromatography mass spectrometry
HPLC	High-performance liquid chromatography
HRESIMS	High-resolution electrospray ionization mass spectrometry
TLC	Thin-layer chromatography
TPC	Total phenolic content
TFC	Total flavonoid content
TPAC	Total phenolic acids content
TTC	Total tannins content
ABTS	2,2′-azino-bis(3-ethylbenzthiazoline-6-sulfonic acid)
FRAP	Ferrous reducing antioxidant potential
DPPH	2,2-diphenyl-1-picrylhydrazyl
CUPRAC	CUPric reducing antioxidant capacity
FALGPA	*N*-[3-(2-furyl)acryloyl]-leu-gly-pro-ala
EGCG	Epigallocatechin gallate
TOF	Time-of-flight
CD_3_OD	Deuterated methanol
DMSO	Dimethyl sulfoxide
CC	Column chromatography
MeOH	Methanol
Et_2_O	Diethyl ether
EtOAc	Ethyl acetate
*n*-BuOH	*n*-butanol
UPW	Ultra-pure water
ACN	Acetonitrile
mp	Melting point
SD	Standard deviation
ANOVA	Analysis of variance
ECM	Extracellular matrix
MMP	Matrix metalloproteinases
hex	Hexose moiety
pent	Pentose moiety
LOD	Limits of detection
LOQ	Limits of detection quantification
SDa	Standard deviation of the response
