# Supplementary material for: Determination of Flavonoids in Selected Scleranthus Species and Their Anti-Collagenase and Antioxidant Potential
Source: Molecules. 2022 Mar 21;27(6):2015. doi: 10.3390/molecules27062015 (PMC8951040; doi:10.3390/molecules27062015)
Supplement: Supplementary file 1 [file molecules-27-02015-s001.zip › molecules-1642633-supplementary.pdf]

molecules

Supplementary Materials

Determination of flavonoids in selected *Scleranthus* species and their anti-collagenase and antioxidant potential

Katarzyna Jakimiuk [1], Jakub W. Strawa [1], Sebastian Granica [2], Marcello Locatelli [3], Angela Tartaglia [3] and Michał Tomczyk [1,*]

[1] Department of Pharmacognosy, Faculty of Pharmacy with the Division of Laboratory Medicine, Medical University of Białystok, ul. Mickiewicza 2a, 15-230 Białystok, Poland; katarzyna.jakimiuk@umb.edu.pl (K.J.); jakub.strawa@umb.edu.pl (J.W.S.); michal.tomczyk@umb.edu.pl (M.T.)

[2] Microbiota Lab, Center for Preclinical Studies, Department of Pharmacognosy and Molecular Basis of Phytotherapy, Faculty of Pharmacy, Medical University of Warsaw, ul. Banacha 1, 02-097 Warsaw, Poland; sgranica@wum.edu.pl (S.G.);

[3] Department of Pharmacy, University of Chieti−Pescara "G. d'Annunzio", Chieti 66100, Italy; m.locatelli@unich.it (M.L.); angela.tartaglia@unich.it (A.T.)

* Correspondence: michal.tomczyk@umb.edu.pl (M.T.); Tel.: +48-85-748-56-94

General experimental procedures

Solvents to extraction and fractionation: petrol, chloroform ($CHCl_3$), methanol (MeOH) ethyl acetate (EtOAc), diethyl ether (Et2O), *n*-butanol (BuOH), were purchased from POCH (Gliwice, Poland). Acetonitrile Optima (ACN), was provided by Fisher Chemical (Loughborough, UK). Ultra-pure water (UPW) was obtained in the Department of Pharmacognosy using the POLWATER DL3-100 system (Kraków, Poland). Natural product reagent A (NA) was obtained from Carl Roth (Karlsruhe, Germany). Phthalic acid, aniline, collagenase from *Clostridium histolyticum* (C0130), sodium chloride (NaCl), calcium chloride ($CaCl_2$), and N-[3-(2-Furyl)acryloyl]-leu-gly-Pro-Ala (FALGPA), Ferric Reducing Antioxidant Power Kit (MAK369), Antioxidant Assay Kit (CS0790) for ABTS assay, Antioxidant Assay Kit (MAK334) for CUPRAC assay, Folin–Ciocalteu reagent, 2,2-Diphenyl-1-Picrylhydrazyl (DPPH), sodium carbonate (Na_2CO_3) were purchased from Sigma-Aldrich (Poole, Great Britain). Formic acid (FA), hydrochloric acid (HCl), acetic acid (AcOH), dimethyl sulfoxide (DMSO), 25% ammonia solution (NH_4OH), ethanol (EtOH) were provided by Carl Roth (Karlsruhe, Germany). Positive control in collagenase assay, epigallocatechin gallate (EGCG), plates for TLC method (silica gel and microcrystalline cellulose), as well as glucose, xylose, rhamnose for TLC analysis were purchased from Merck KGaA (Darmstadt, Germany), and uronic acids (glucuronide acid, galacturonide acid) were purchased from Cayman Chemical (Ann Arbor, MI, USA). Sephadex LH-20 to column chromatography (CC) was provided by GE Healthcare Bio-Sciences AB (Uppsala, Sweden). The qualitative assessment was carried out with an Ultimate 3000 series system coupled with an Amazon SL (Bruker, Bremen, Germany) ion trap mass spectrometer. Quantitative analyses were conducted using an Agilent Technologies 1260 Infinity chromatography system equipped with a 1290 Infinity photodiode array detector (Santa Clara, CA, USA). Preparative HPLC analyses were performed on a Shimadzu instrument (Columbia, MD, USA) with SPD-10ATvp detector, an LC20-AP pumps, an LC-10AF autosampler, and an FRC-10A fraction collector. UV spectra were carried out on an Analytic Jena SPECORD 200 Plus instrument (Jena, Germany). Melting points were obtained using the Stuart™ SMP10 apparatus (Bibby Sterlin, United Kingdom). NMR spectra were recorded on a Thermo Fisher Scientific Bruker Advance II 400 spectrometer (Waltham, MA, USA) at 400 MHz in deuterated methanol (CD3OD). Optical rotation of compound **6** was measured with JASCO P-2000 (Tokyo, Japan). Anti-collagenase and antioxidant assays were performed on BioTek Instruments microplate spectrophotometer EPOCH 2 (Oxfordshire, UK).

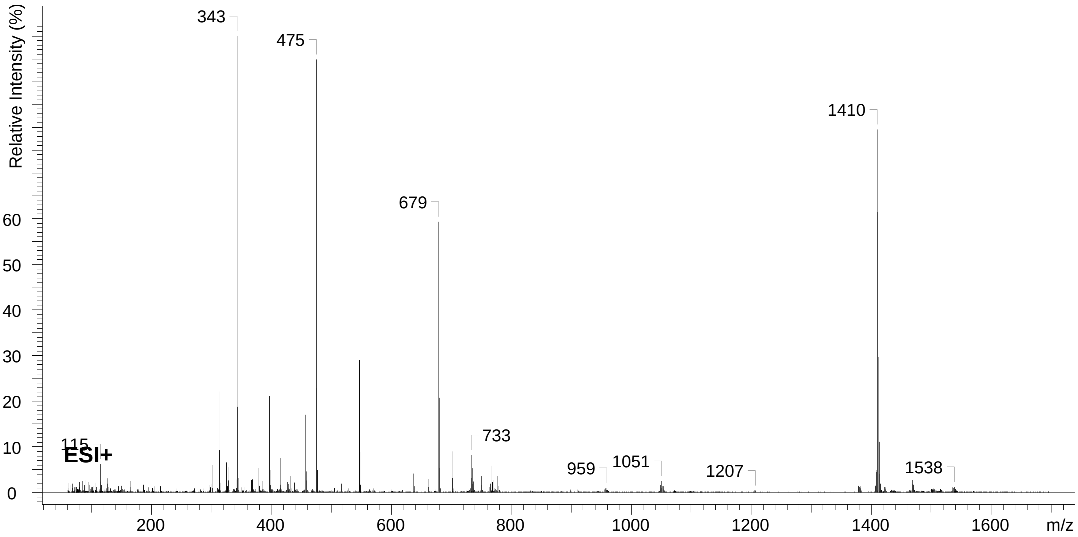

Figure S1. Product ion scan in positive mode of compound **6** (300 V).

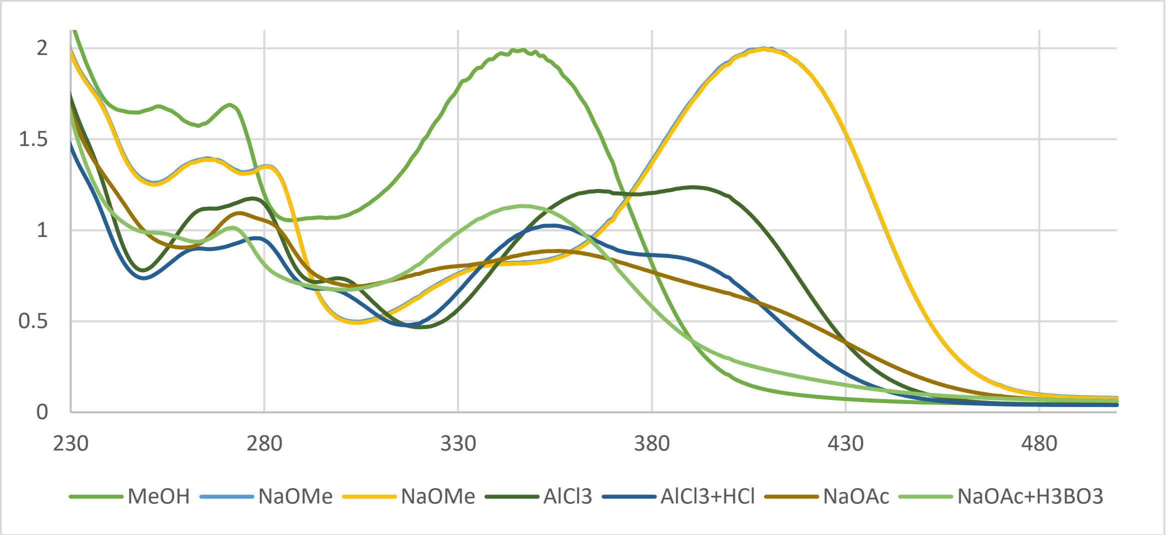

Figure S2. UV spectrum of compounds **6**.

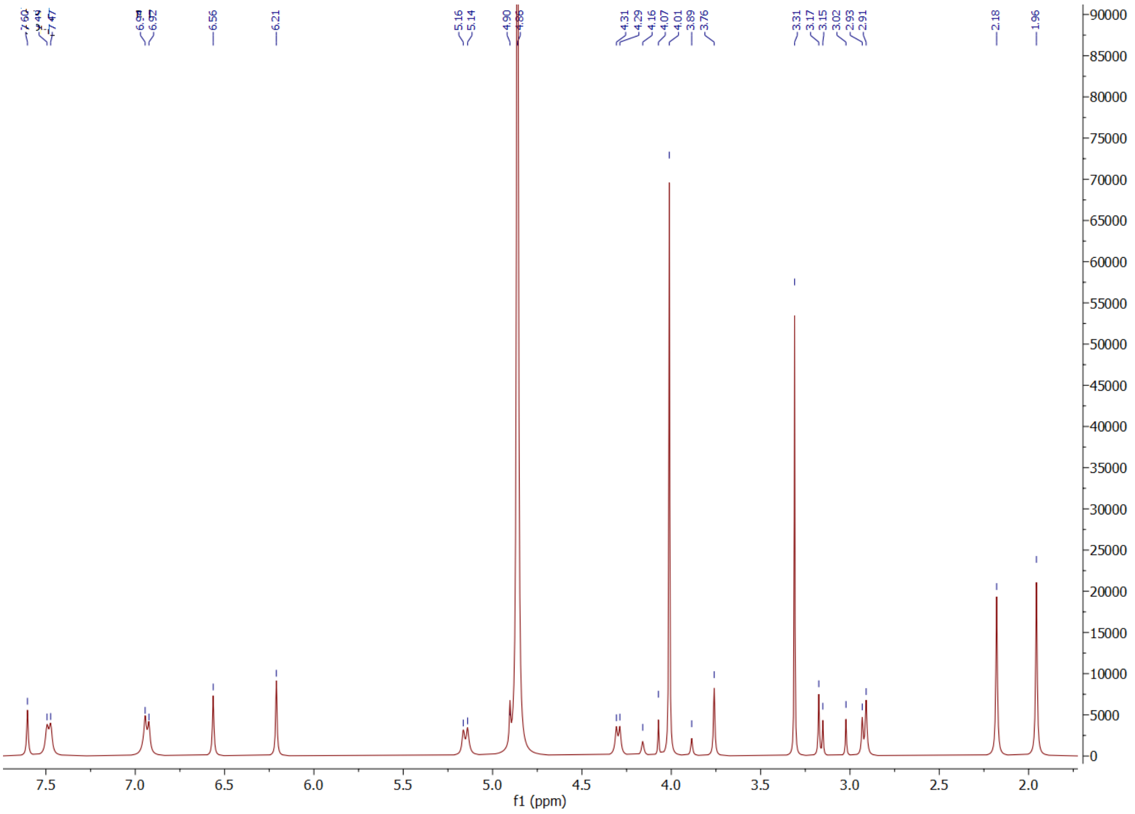

Figure S3. ^{1}H NMR spectrum (400 MHz) of compound **6** in CD_3OD.

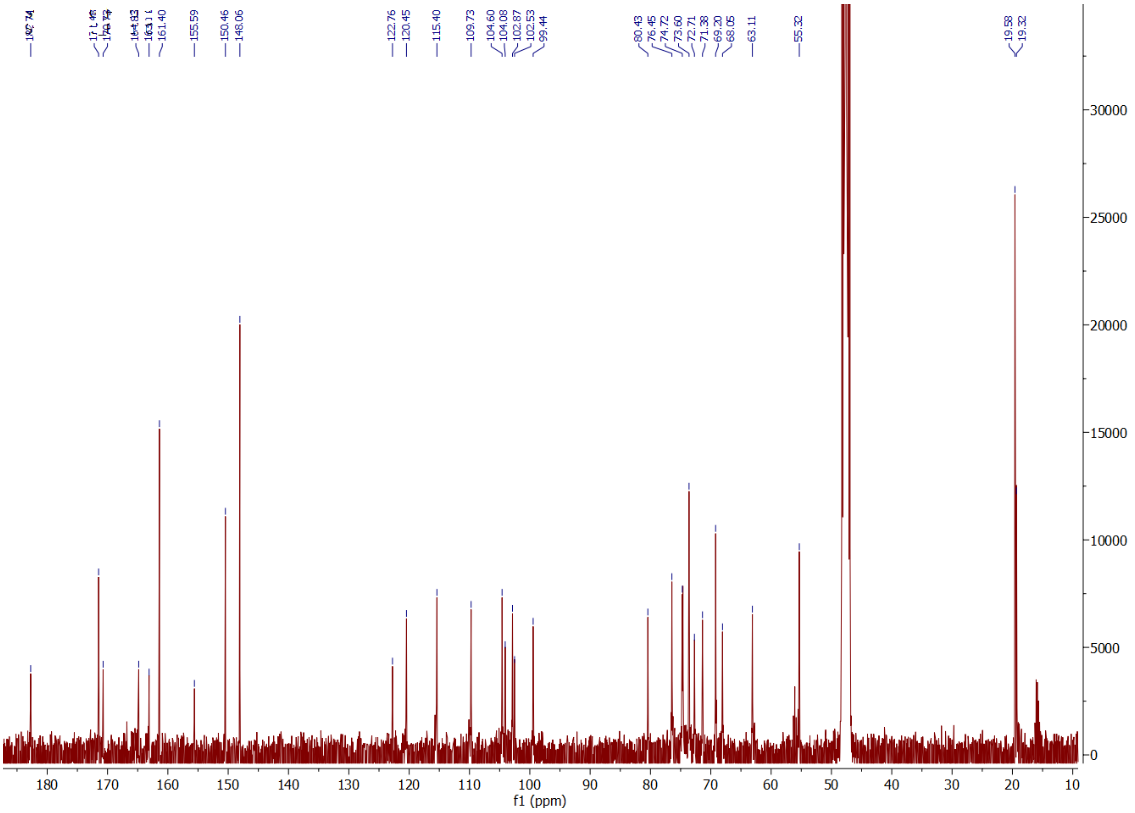

Figure S4. ^{13}C NMR spectrum (400 MHz) of compound **6** in CD_3OD.

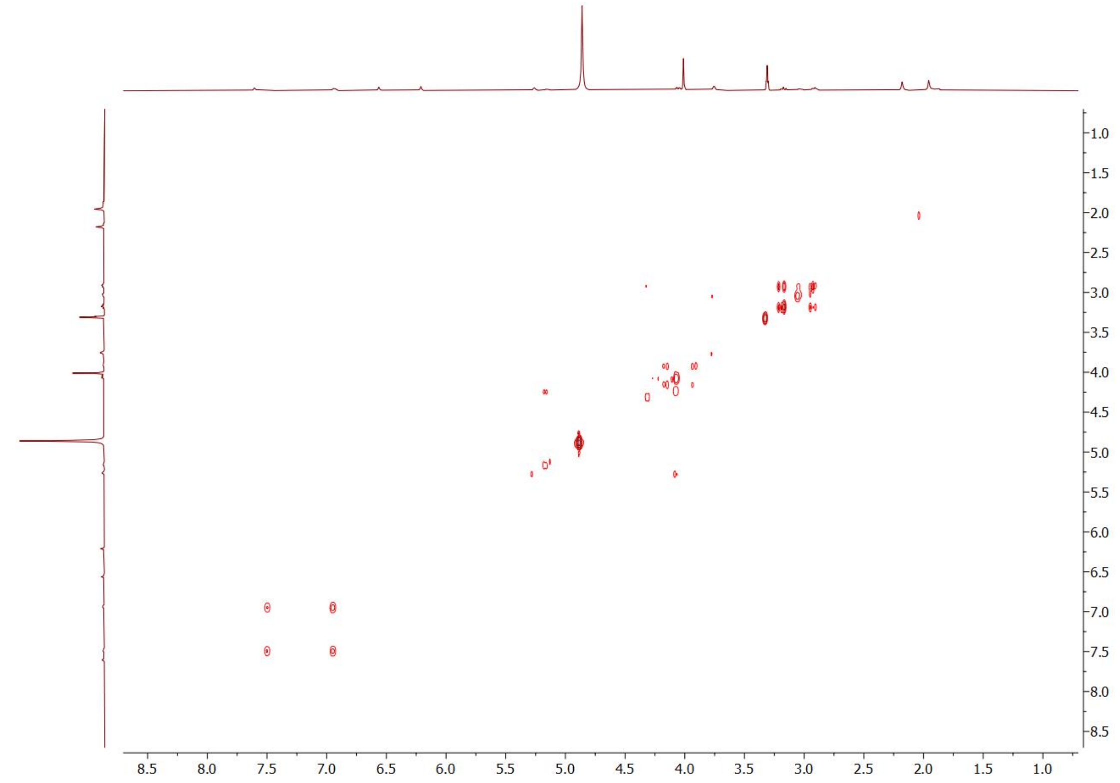

Figure S5. ^1H-^1H COSY spectrum of compound **6** in CD_3OD.

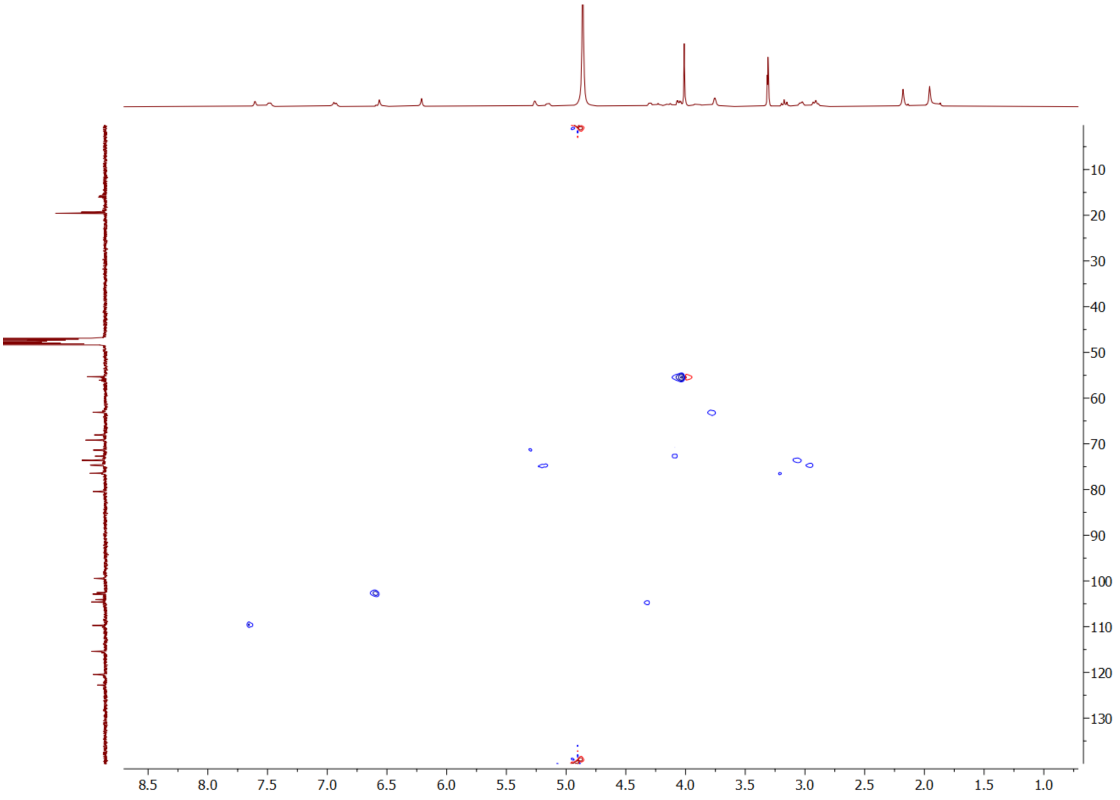

Figure S6. HSQC spectrum of compound **6** in CD_3OD.

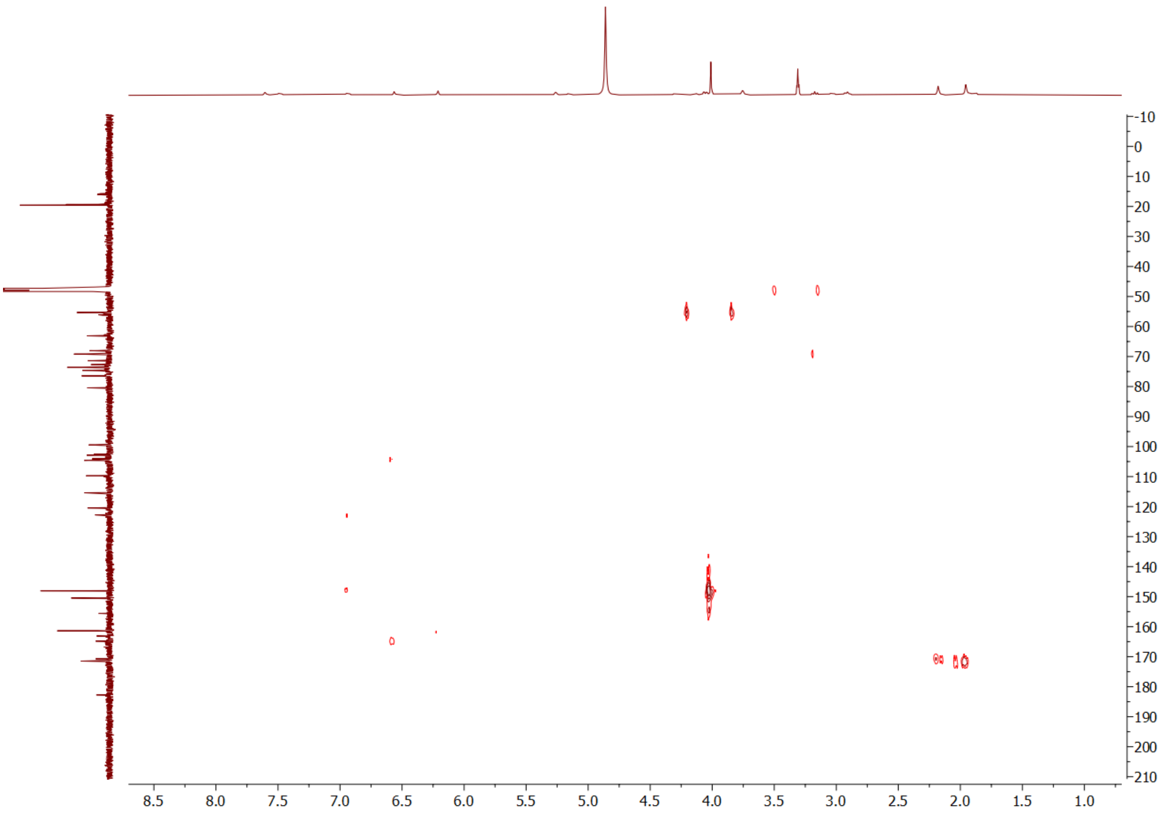

Figure S7. HMBC spectrum of compound **6** in CD_3OD.

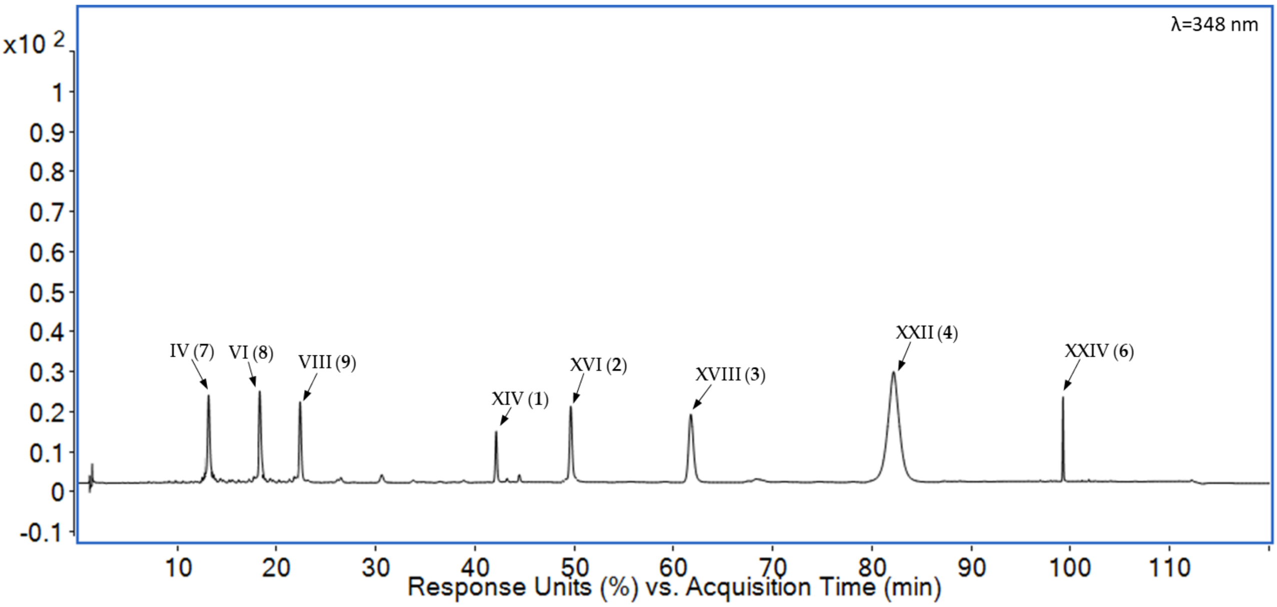

Figure S8. UV-VIS chromatogram (λ = 348nm) of separated compounds **1-4, 6-9** obtained by LC-PDA–MS.

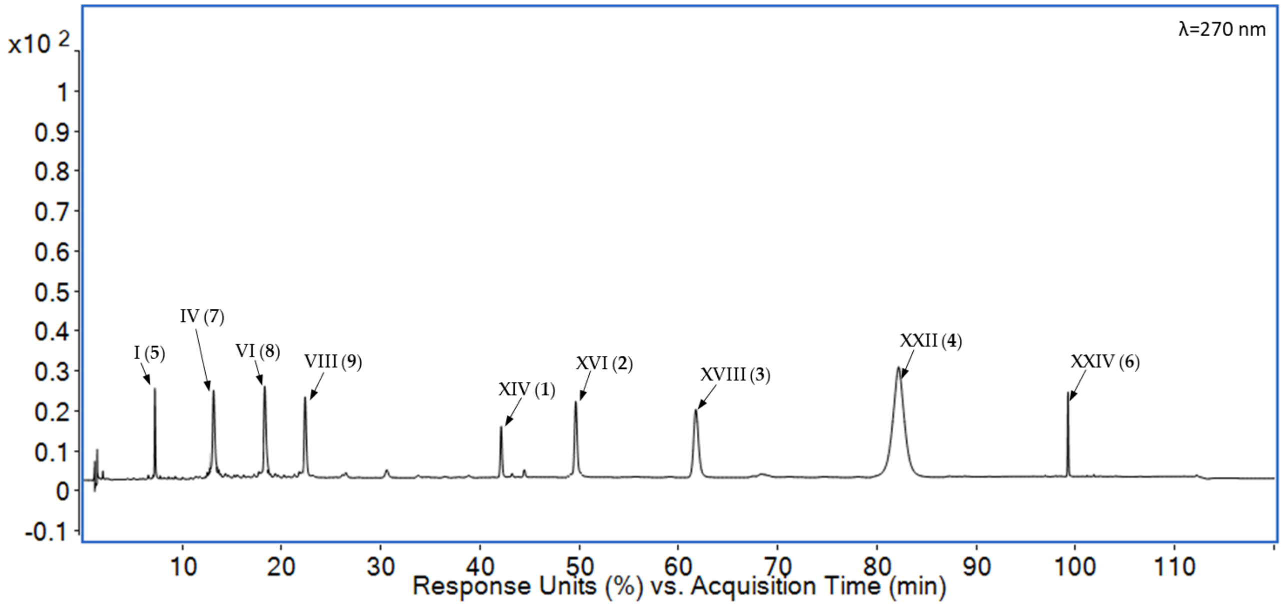

Figure S9. UV-VIS chromatogram (λ = 270 nm) of separated compounds **1-9** obtained by LC-PDA–MS.

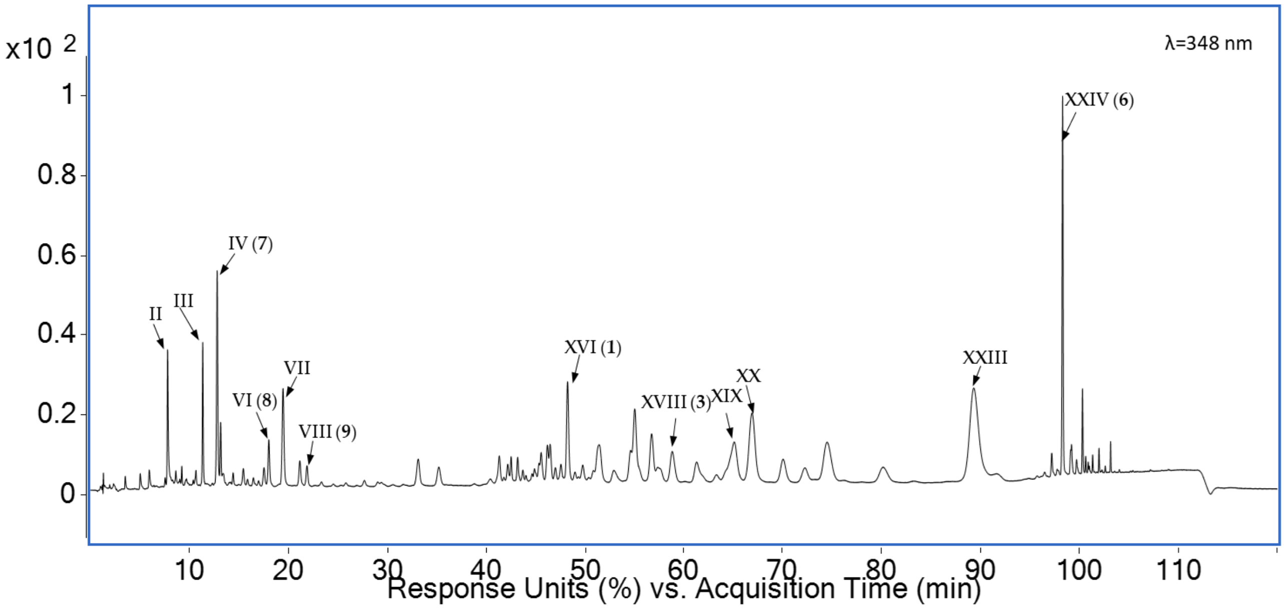

Figure S10. The qualitative assessment of **SA1**. UV-VIS chromatogram (λ = 348 nm) obtained by LC-PDA–MS.

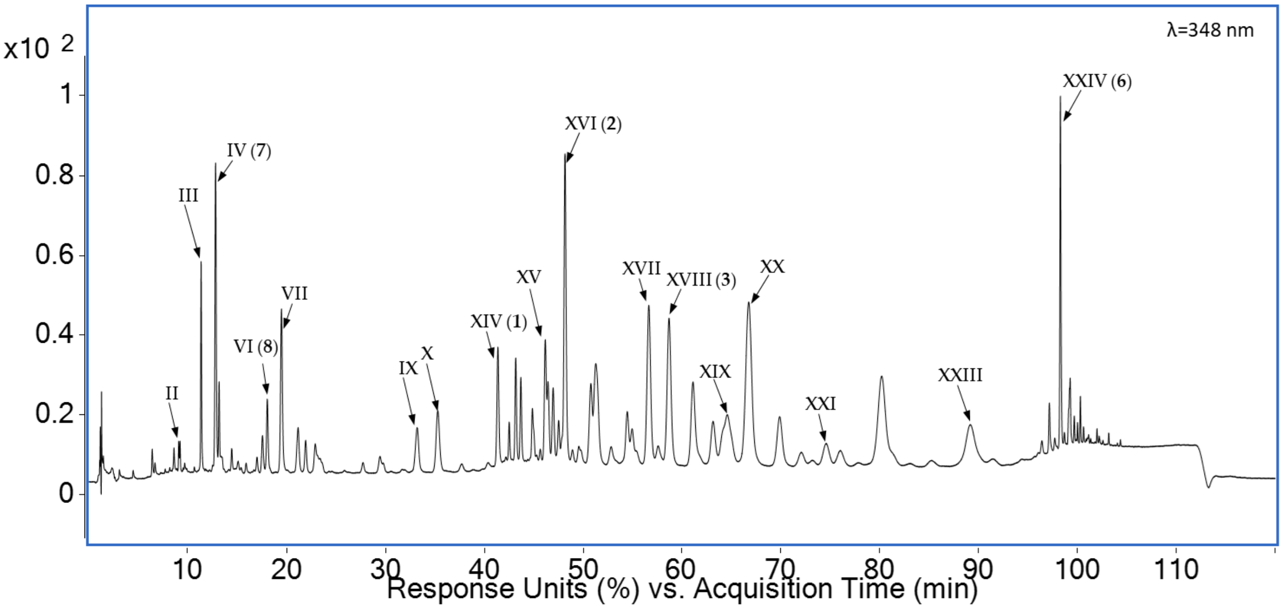

Figure S11. The qualitative assessment of **SA2**. UV-VIS chromatogram (λ = 348 nm) obtained by LC-PDA–MS.

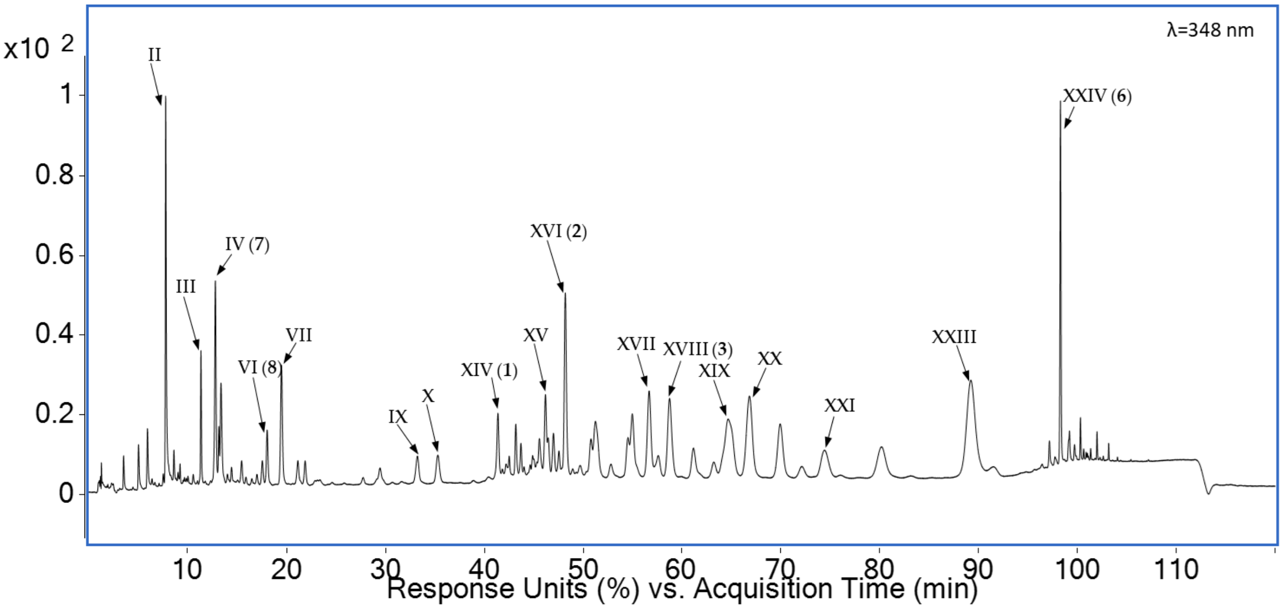

Figure S12. The qualitative assessment of **SA3**. UV-VIS chromatogram (λ = 348 nm) obtained by LC-PDA–MS.

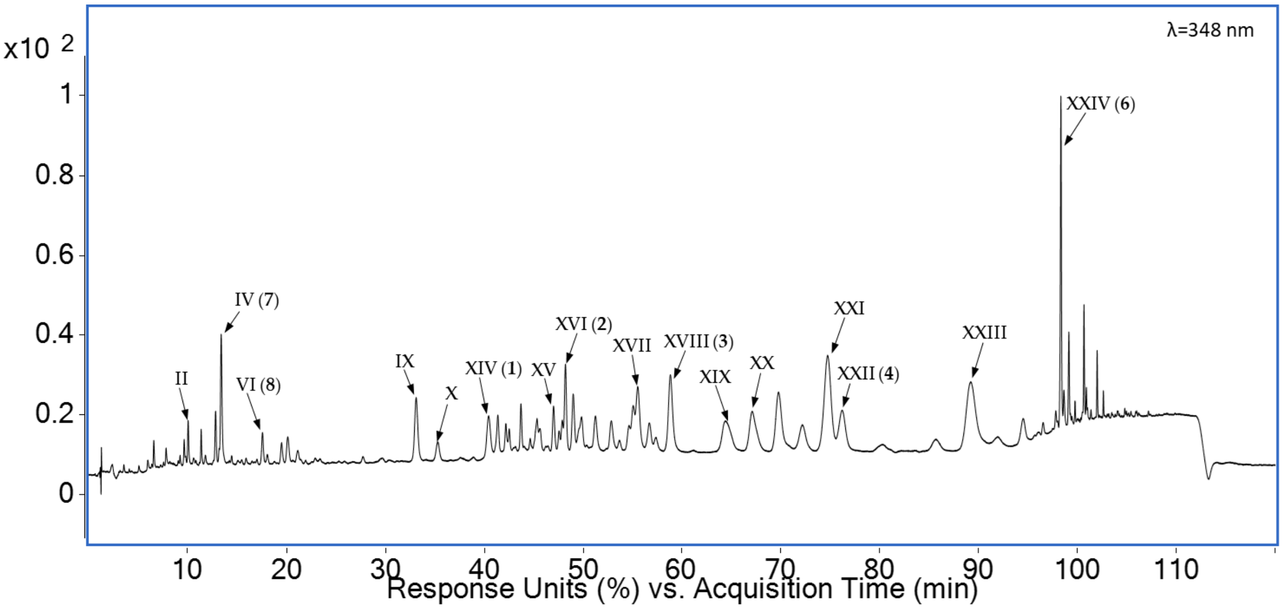

Figure S13. The qualitative assessment of **SA4**. UV-VIS chromatogram (*λ* = 348 nm) obtained by LC-PDA–MS.

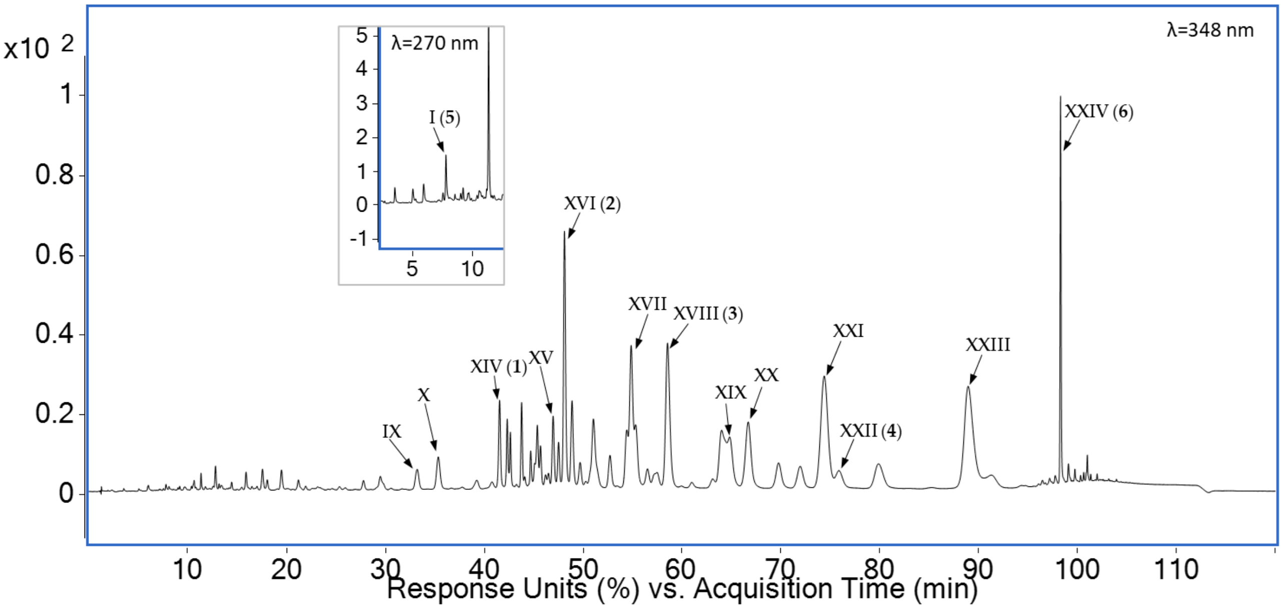

Figure S14. The qualitative assessment of **SA5**. UV-VIS chromatogram (*λ* = 348 nm and 270 nm) obtained by LC-PDA–MS.

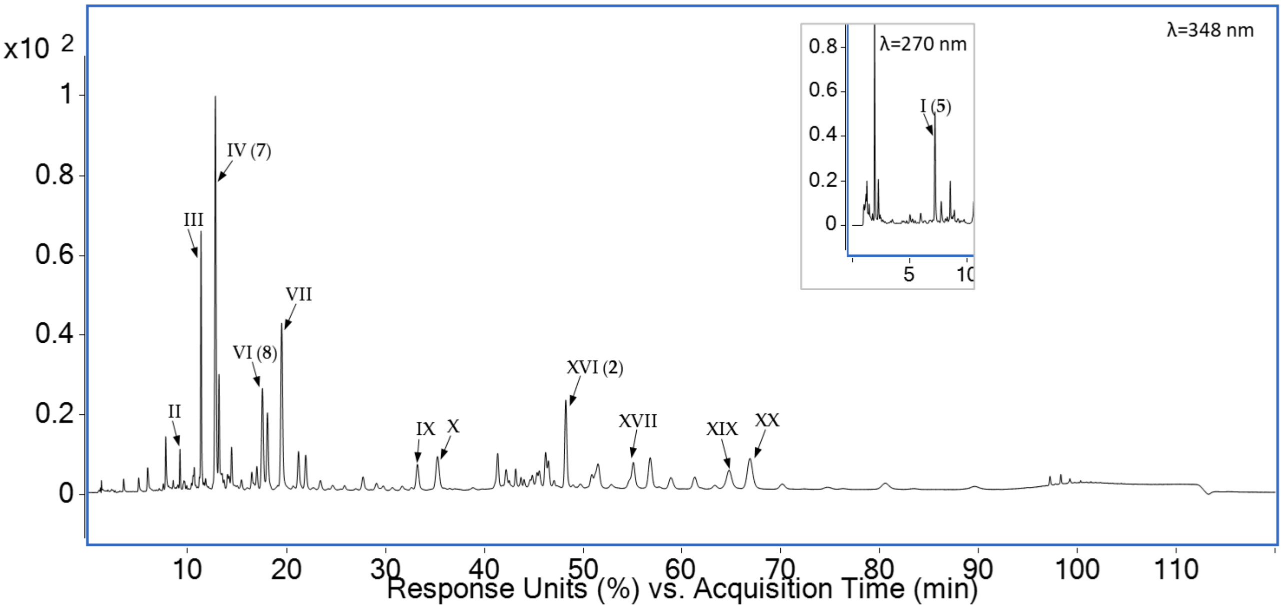

Figure S15. The qualitative assessment of **SA6**. UV-VIS chromatogram (*λ* = 348 nm and 270 nm) obtained by LC-PDA–MS.

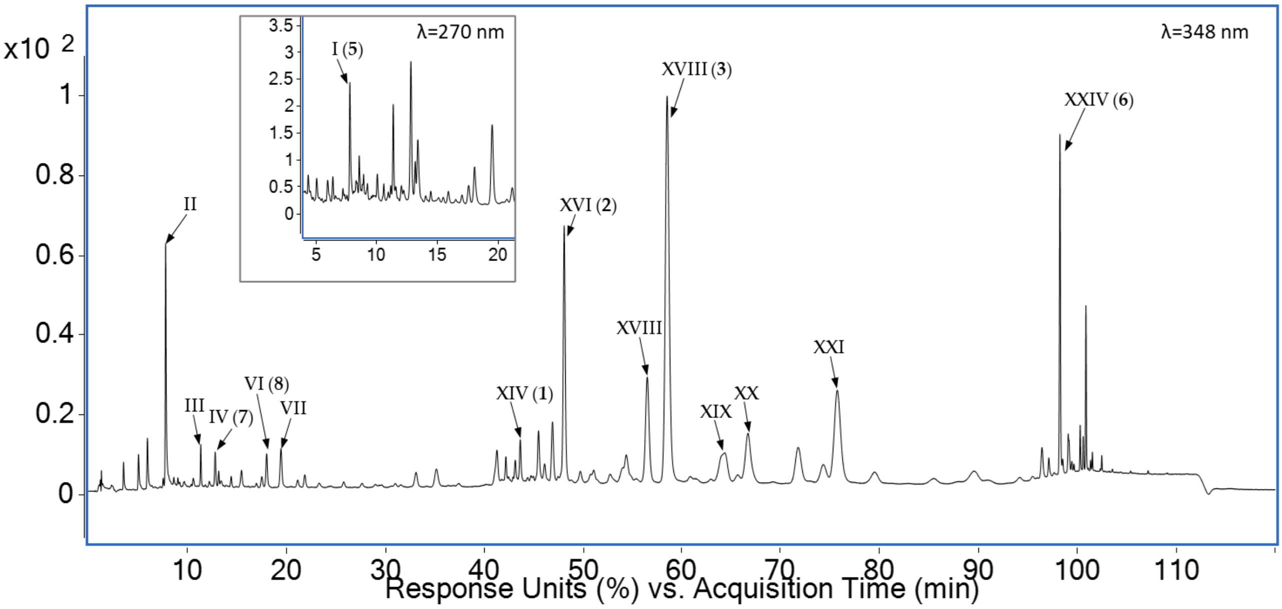

Figure S16. The qualitative assessment of **SP1**. UV-VIS chromatogram (*λ* = 348 nm and 270 nm) obtained by LC-PDA–MS.

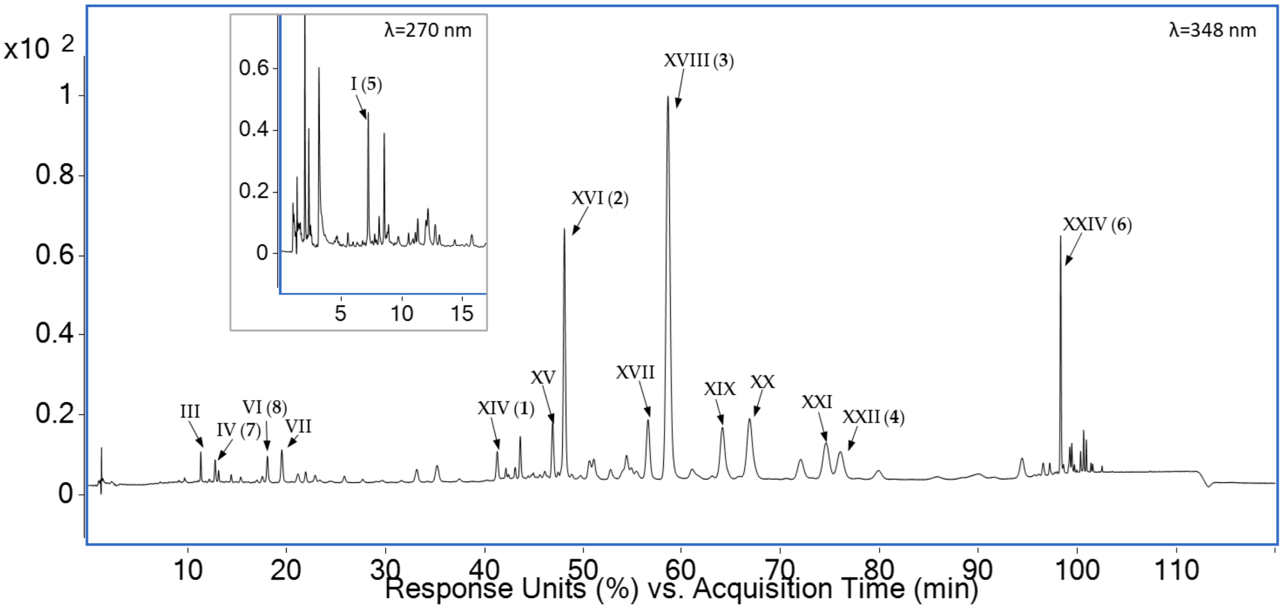

Figure S17. The qualitative assessment of **SP2**. UV-VIS chromatogram (λ = 348 nm and 270 nm) obtained by LC-PDA–MS.

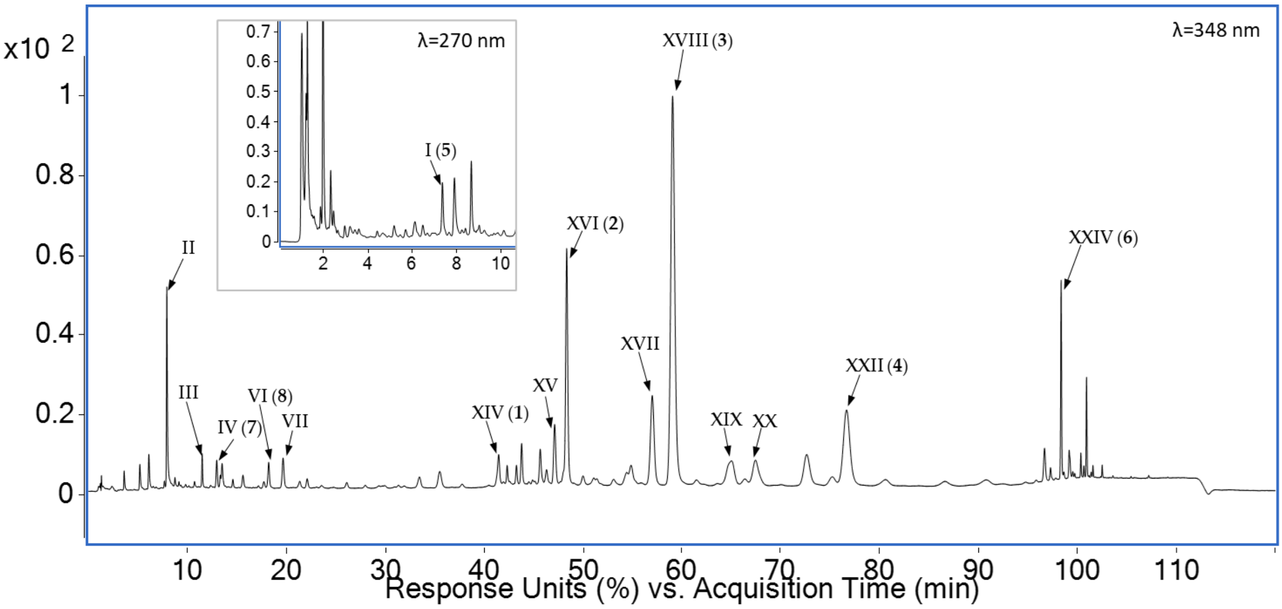

Figure S18. The qualitative assessment of **SP3**. UV-VIS chromatogram (λ = 348 nm and 270 nm) obtained by LC-PDA–MS.

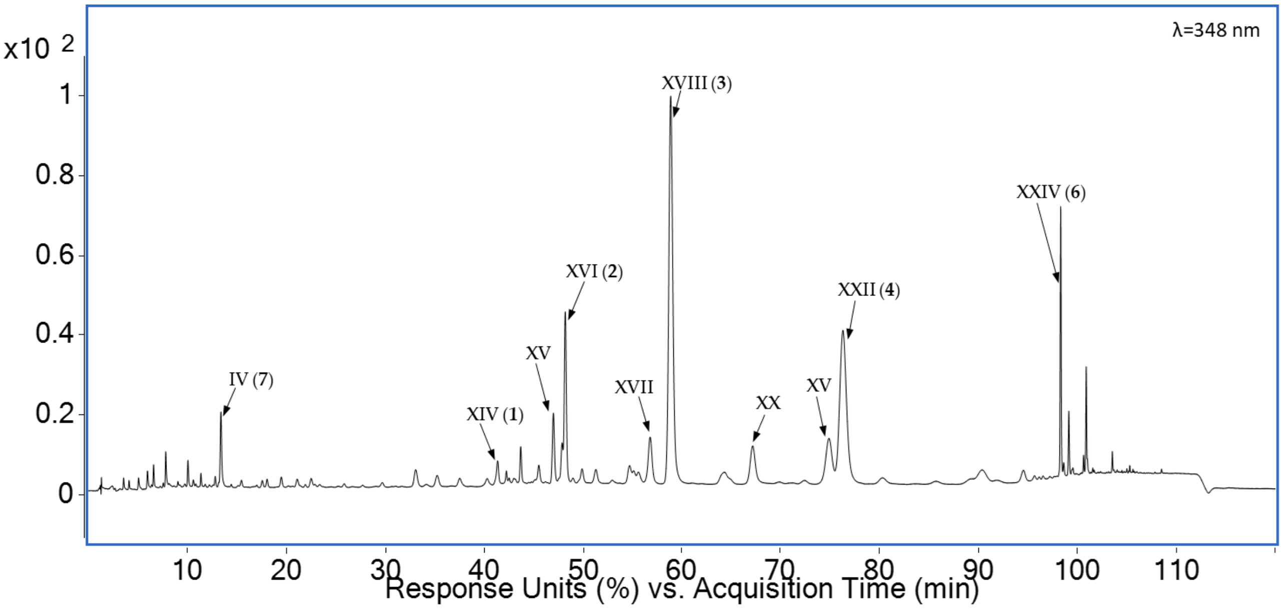

Figure S19. The qualitative assessment of **SP4**. UV-VIS chromatogram (λ = 348 nm) obtained by LC-PDA–MS.

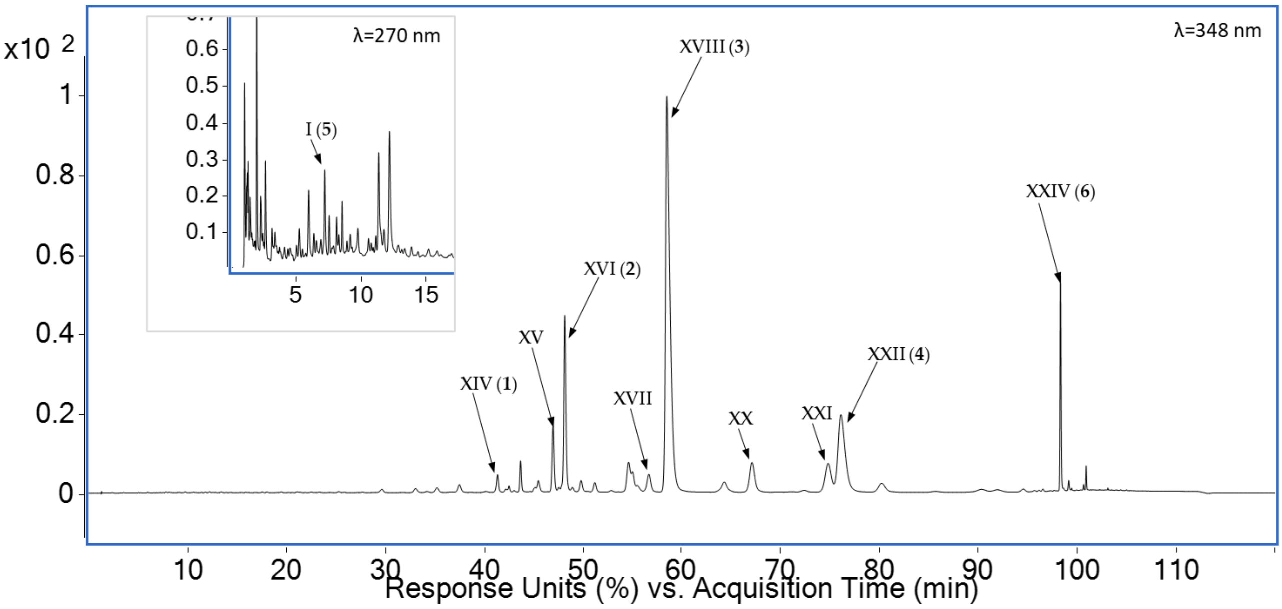

Figure S20. The qualitative assessment of **SP5**. UV-VIS chromatogram (λ = 348 nm and 270 nm) obtained by LC-PDA–MS.

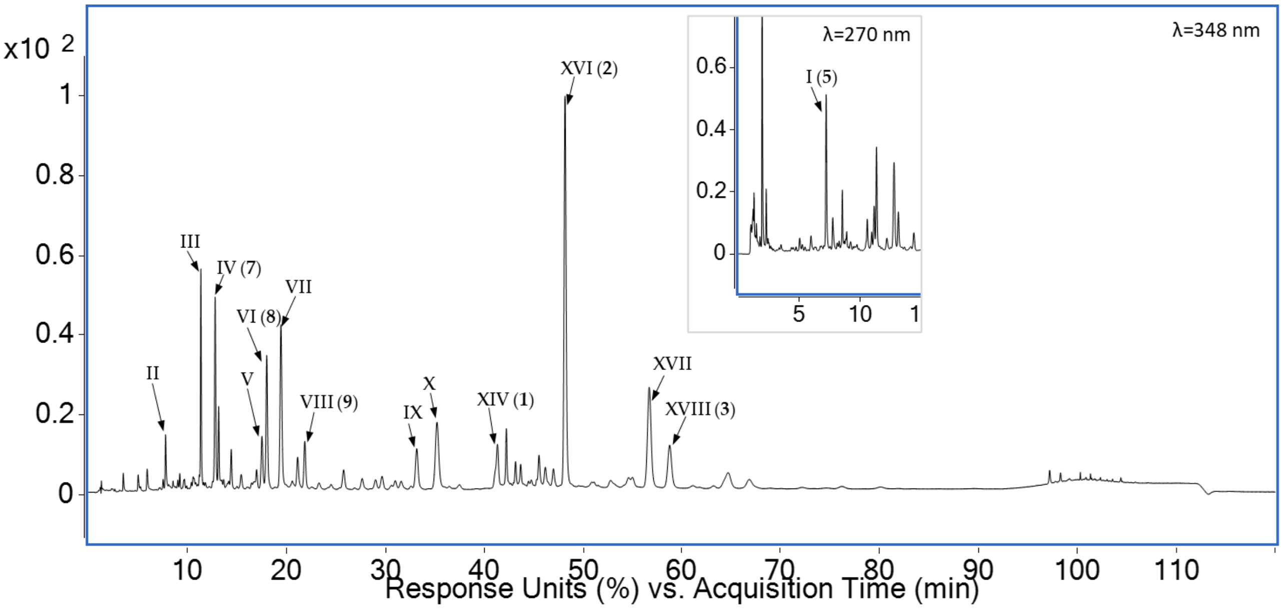

Figure S21. The qualitative assessment of **SP6**. UV-VIS chromatogram (λ = 348 nm and 270 nm) obtained by LC-PDA–MS.
